# Evolution of the Metazoan Protein Domain Repertoire Revealed by a Birth-Death-Gain Model

**DOI:** 10.1007/s00239-025-10286-0

**Published:** 2025-12-29

**Authors:** Yuting Xiao, Maureen Stolzer, Larry Wasserman, Dannie Durand

**Affiliations:** 1 Biological Sciences, Carnegie Mellon University, Pittsburgh, PA 15213 USA; 2 Ray and Stephanie Lane Computational Biology, Carnegie Mellon University, Pittsburgh, PA 15213 USA; 3 Statistics and Data Science, Machine Learning, Carnegie Mellon University, Pittsburgh, PA 15213 USA

**Keywords:** Protein domain, Birth-Death-Gain model, Evolutionary rates

## Abstract

Reconstruction of the ancestral protein repertoire offers valuable insights into the tempo and mode of protein content evolution, but can be highly sensitive to model choice. We used a phylogenetic Birth-Death-Gain model to investigate the evolution of the metazoan protein domain repertoire. Domains, protein modules with a distinct structure and function, represent the basic components of protein repertoire.  Given a species tree and a census of protein domain families in present-day species, we estimated the most likely rates of domain family origination, duplication, and loss. Rates were allowed to vary across species lineages and domain families, decoupling these factors. Statistical hierarchical clustering of family-specific rates reveals groups of domains evolving in concert. Moreover, we observe a strong and significant association between family rate and family function.  Interestingly, families with functions associated with metazoan innovations tend to have the fastest rates. We further inferred the expected ancestral domain content and the history of domain family gains, losses, expansions, and contractions in each species lineage. Our analysis reveals an ongoing process of domain family replacement and resizing, consistent with extensive remodeling of the protein domain repertoire. This stands in contrast to recent reports of widespread loss during metazoan evolution, which were obtained with more constrained models. The use of a powerful, probabilistic Birth-Death-Gain model reveals an unexpected level of genomic plasticity.

**Supplementary Information:**

The online version contains supplementary material available at 10.1007/s00239-025-10286-0.

## Introduction

**Fig. 1:**
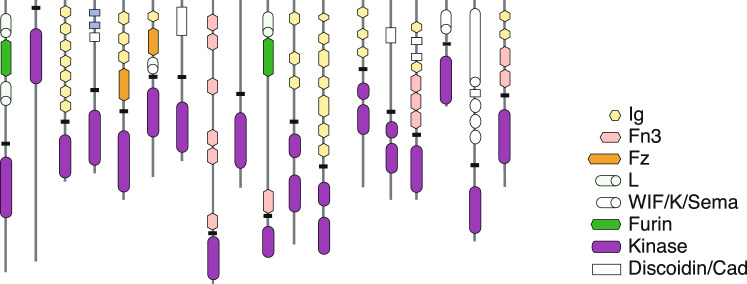
Domain architectures in a complex multidomain protein family, the receptor subfamily of the human protein tyrosine kinases, adapted from Robinson et al. (2000)

The expanding wealth of genome sequences from metazoa and their closest unicellular relatives is driving a growing body of work investigating the relationship between the evolution of the metazoan protein-coding repertoire and innovations on other levels of biological organization. Phylogenetic comparative analysis is being used to probe the genetic basis of metazoan innovation broadly (Wang et al. 2012; Technau and Schwaiger 2015; Paps and Holland 2018; Fernández and Gabaldón 2020; Guijarro-Clarke et al. 2020; Zmasek and Godzik 2011, 2012; Domazet-Lošo et al. 2024; Suga et al. 2013; Grau-Bové et al. 2017; López-Escardó et al. 2019; Richter et al. 2018), or with a focus on specific systems, including the bilaterian body plan (Heger et al. 2020), the metazoan nervous system (Emes et al. 2008; Ryan and Grant 2009; Liebeskind et al. 2015, 2016), and membrane proteins (Nam et al. 2015; Attwood et al. 2017). Protein domains, domain combinations, and full-length proteins provide three related, but distinct views of the protein repertoire encoded in a genome (Fig. 1). While most studies of the protein coding repertoire have focused on full length protein families, domains (Zmasek and Godzik 2011; Moore and Bornberg-Bauer 2012; Wang et al. 2012; López-Escardó et al. 2019; Suga et al. 2013) and domain combinations (Grau-Bové et al. 2017; Zmasek and Godzik 2012) have also been considered.

Ancestral state reconstruction proceeds from a set of phylogenetic profiles, one for each family, representing the number of copies of the family encoded in each present day genome. Ancestral reconstruction methods infer the protein coding repertoire in ancestral genomes and the changes along the branches of the species tree that best explain the present-day profiles with respect to a set of evolutionary assumptions. This provides a basis for inferring the phenotypic complexity, habitat, and ecology of ancestral species. Determining when genes associated with major phenotypic innovations first appeared reveals the timing of those innovations and whether they arose multiple times independently. Ancestral reconstruction also sheds light on the tempo and mode of evolution and the relative importance of gene duplication, horizontal transfer, and loss in various lineages. For example, reports of a preponderance of loss in Metazoa (Guijarro-Clarke et al. 2020; Domazet-Lošo et al. 2024) and other taxonomic lineages (Qiu et al. 2018; Bowles et al. 2020; Zmasek and Godzik 2011) suggest the importance of genome reduction in evolution (Wolf and Koonin 2013; Albalat and Cañestro 2016).

The results of such studies depend, often crucially, on methodological choices. Parsimony methods infer ancestral states that minimize the weighted sum of gains and losses. There are numerous parsimony variants that differ in their evolutionary assumptions and their propensity to invoke parallel gains, parallel losses, or reversals to resolve homoplasy (Swofford and Maddison 1987). Dollo parsimony, which is widely used in comparative genomic studies, applies the further restriction that a character may only be gained once (Farris 1977). As a consequence, Dollo parsimony allows parallel losses, but not parallel gains or reversals, and hence implies that all families were present in the common ancestor of the species that harbor the families. Probabilistic methods that do not require minimal change implement parameterized mathematical models that embody a set of evolutionary assumptions and seek the parameter values and ancestral states that maximize the likelihood of present-day families (Harmon 2019; Pagel 1999). Probabilistic ancestral reconstruction methods also embody a range of evolutionary models. The underlying assumptions may be explicit features of the model or result implicitly from simplifying assumptions required for efficient calculation of the likelihood.

**Fig. 2:**
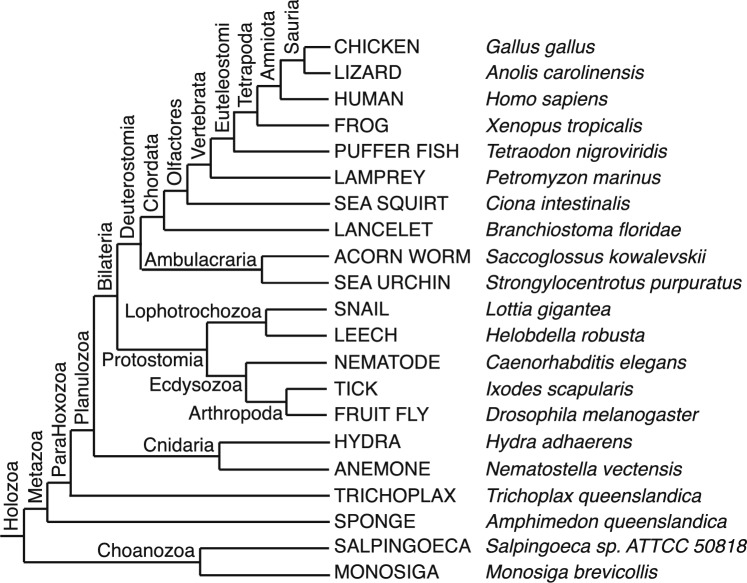
The undated phylogeny of the 21 holozoan species used in our analysis, adapted from Philippe et al. (2009). Branch lengths were selected for visual clarity and do not represent evolutionary change. For reference, a dated species tree from TimeTree (Kumar et al. 2022) is provided in A1b

In this study, we apply a probabilistic, Birth-Death-Gain (BDG) model, implemented in the COUNT software package (Csűrös 2010; Csűrös and Miklós 2009), to investigate the evolution of the protein domain repertoire encoded in the genomes of 21 species spanning the evolutionary trajectory of the metazoa and their closest unicellular relatives (Fig. 2). The evolutionary events that modify the protein coding repertoire—birth (i.e., duplication of a domain already encoded in the genome), death (loss of a domain), and gain (de novo domain acquisition)—are explicit parameters of the model. In the context of domain family evolution, domain duplication comprises any evolutionary process that creates a new copy of a domain, including both gene duplication and domain duplication. Similarly, domain deletion can result from the loss of a domain or the loss of an entire gene.

COUNT’s design reflects a general evolutionary model. The inclusion of a gain event in the model relaxes the assumption that all families observed today were present in the common ancestor and allows for multiple independent acquisitions of a family via horizontal transfer. In addition, COUNT allows rates to vary across both species tree branches and across domain families. Each event rate is the product of a branch-specific rate and a unit-less family-specific rate factor. Family-specific rate factors (or just rates) modulate the underlying branch rate to accommodate families evolving at a range of rates. This architecture makes it possible to probe rate variation across families, independent of branch-specific effects. The flexibility of the COUNT model has the potential to reveal plasticity, if such exists, that could not be uncovered with a more constrained model. We investigate the evolution of the protein domain repertoire through the lens of these model properties.

Here, we apply COUNT to reconstruct the evolutionary history of 1283 metazoan protein domain families. Given a species tree and present-day domain counts, COUNT infers birth, death, and gain rates that maximize the likelihood of the present day repertoire, as well as the probability of ancestral states and state changes on the tree. We take advantage of COUNT’s event model to examine how quickly families evolve, relative to each other and independent of branch-specific variation. Most ancestral reconstruction models are not able to infer the family-specific and branch-specific components of evolutionary event rates, separately. Prior work on domain gain and loss rates focuses on rate variation across branches, only (Nasir et al. 2014; Coban et al. 2025; Moore and Bornberg-Bauer 2012). To our knowledge, family-specific rate data in metazoa has rarely, if ever, been examined.

We compare domain family rates and domain function using a publicly available domain-specific functional ontology (Vogel et al. 2004, 2005) and observe a statistically significant association between the function of a family and its evolutionary rates. Notably, families with metazoa-associated functions tend to have the fastest rates of change. Further, statistical hierarchical clustering of the family-specific rates reveals significant substructure in the distribution of rates, consistent with groups of families following similar evolutionary trajectories.

We next use the COUNT likelihood model to estimate the size of the domain repertoire in ancestral genomes, as well as the expected number of families that were gained and lost on each branch. Monotonic genome expansion or reduction is relatively rare in these reconstructions. Rather, we see continual turnover in the composition of the ancestral repertoire. In some lineages, newly gained families take the place of recent family extinctions. In other lineages, decreases in the number of families are offset by size increases in the remaining families. Only 6 lineages, out of 40, manifest *bona fide* genome streamlining with simultaneous reduction in the number of families and in the number of copies per family.

The modest evidence for genome reduction in our reconstruction is surprising given recent reports of widespread loss in metazoa (Guijarro-Clarke et al. 2020; Domazet-Lošo et al. 2024). Could this discrepancy be due to differences in the model assumptions, since unlike Dollo parsimony, BDG does not exert a preference for minimal change and entails no a priori preference for losses versus gains? We test this hypothesis by reanalyzing the 1283 metazoan domain families using Dollo parsimony. Dollo yields a four-fold increase in the number of losses per gain, confirming our suspicions.

The results presented in the following sections provide a cautionary example of model choice resulting in dramatically different outcomes. The reconstructions obtained with Dollo parsimony and the probabilistic BDG model are not merely quantitatively different, but support fundamentally different views of the forces driving evolutionary change in metazoa.

## Methods

### Data

The Birth-Death-Gain analysis was carried out using domain data from the genomes of 21 holozoan species (19 metazoa and two choanoflagellates) and three outgroups: *Neurospora crassa*, *Dictyostelium discoideum*, and *Arabidopsis thaliana*. Genomes used in this analysis were chosen to probe major metazoan clades, while also considering genome quality. In addition, taxa were selected to avoid very short or very long branches along the backbone of the phylogeny to minimize artifacts and computational problems associated with extreme branch lengths. For this reason, ctenophores are not represented in this study.

The species phylogeny used in this study (Fig. A1) is based on that of Philippe et al. (2009). Early metazoan branching order is controversial. In the phylogeny used in this work, sponge and Planulozoa are sister taxa, where the planulozoan clade comprises Cnidaria and Bilateria (Lartillot and Philippe 2008; Philippe et al. 2009; Telford et al. 2015). While the sister clade to sponge is often referred to as Eumetazoa, we do not use this terminology because the term “Eumetazoa” implies a clade that includes ctenophores, which are not represented in our data set. Uncertainty in the tree topology can impact the phylogenetic BDG analysis. The placement of Trichoplax with respect to Cnidaria and Bilateria has also been called into question, with some analyses placing Trichoplax and Cnidaria as sister taxa (Simakov et al. 2020; Laumer et al. 2018). However, other recent work (Laumer et al. 2019; Simion et al. 2017; Najle et al. 2023) continues to support the topology used here (Figs. 2 and A1).

Domain annotations in these 24 genomes were obtained from the SUPERFAMILY database (Oates et al. 2015), which classifies protein domains based on structural and evolutionary relationships using the SCOP (Structural Classification of Proteins) framework (Andreeva et al. 2020; Pandurangan et al. 2019). The SUPERFAMILY classification is hierarchical, with domain models corresponding to SCOP families and SCOP superfamilies. Members of the same superfamily share a structural core, but sequence similarity within the superfamily may be low. We used these top-level assignments for all domain families in our dataset. Since only one level of the hierarchy was used, we use the term “domain family” (and not “superfamily”) throughout this manuscript.

For each of the 24 genomes, domain annotations of protein-coding sequences corresponding to the longest transcript per gene were downloaded from the SUPERFAMILY database, version 1.75 (Oates et al. 2015; Pandurangan et al. 2019). Domain family counts for each species were then tabulated from this data, resulting in 1434 distinct families. The largest 41 families (top $2.5\%$, with more than 3300 instances) were removed before processing to avoid inferring artifactually large duplication rates; 39 of these are encoded in multiple copies (i.e., are multicopy families) in all 21 holozoan species. The remaining 1393 families served as input for the Birth-Death-Gain model described below.

### Phylogenetic Birth-Death-Gain (BDG) Model

We investigated the evolution of domain family sizes using the phylogenetic BDG model implemented in the COUNT software package (Csűrös 2010). The model takes as input an undated species tree, $T = (V, L, B)$, with nodes *V*, leaves $L \subset V$, branches *B*, and a matrix of present-day family sizes. Each family is represented by a phylogenetic profile, a vector of length $\vert L \vert$, where the element at position *S* contains the number of instances of the family encoded in the genome of species $S \in L$. COUNT models family size evolution as a continuous-time Markov process. Family size increases with probability $(\kappa + \lambda n)$ and decreases with probability $\mu n$, where *n* is the family size and $\lambda$, $\kappa$, and $\mu$ are the instantaneous rates of birth, gain, and death events, respectively. The probability that a domain duplication will occur increases with the number of domains in the family, whilst the probability of a gain is independent of family size. The birth-death-gain process on branch $b \in B$, runs for a duration $t_b$. A birth-death model ($\kappa = 0$) entails the implicit assumption that all families that are observed in at least one present-day species were present in their common ancestor. A non-zero gain rate allows for origination of novel domain families in any lineage of the species tree. Family sizes in the common ancestor (i.e., the root of *T*) are assumed to follow a Poisson distribution with mean, $\phi$.

Evolutionary rates in COUNT consist of a branch-specific rate modulated by a family-specific rate factor. The birth, death, and gain rates are the product of a branch-specific rate, a family-specific rate, and a family-specific scaling factor, $\sigma _f$. Thus, the birth rate for family *f* on branch $b \in B$ is $\lambda = \sigma _f \lambda _f \lambda _b$. The death and gain rates are defined analogously. Branch-specific variation allows for different values of the duration ($t_b$) and the birth and gain rates, $\lambda _b$ and $\kappa _b$ on each branch. Rates and branch lengths are scaled such that the branch-specific loss rate is one.

COUNT models rate variation across species tree lineages, input families, or both. COUNT takes an undated species phylogeny as input and uses an internally defined time scale. The branch-specific parameters are defined relative to an internally defined time-scale that is unrelated to external time units (e.g., millions of years). Thus, branch-specific rates are consistent relative to each other and relative to the branch length, $t_b$. Those branch-specific rates are modulated by family-specific rate factors, which determine the evolutionary rates of families relative to each other. Family-specific rate factors adjust the branch-specific rates, resulting in a range of values clustered around the branch-specific rate.

Parameter variation across families is modeled as a discretized gamma distribution with *C* categories, where the value for each category is the mean of the corresponding quantile. Each parameter value for family *f* is represented as a weighted sum over the category values for that parameter, where the weights are estimated from prior probabilities as discussed in greater detail, below. Guided by prior empirical evaluation of the impact of model choice on COUNT performance (Stolzer et al. 2014), in this study we used both branch-specific variation and family-specific variation with $C=2$ categories (i.e. a fast rate and a slow rate), for all four parameters $\{\sigma _f, \kappa _f, \lambda _f, \mu _f\}$.

The amount of change on branch *b* is primarily determined by the branch-specific rates and the duration of the process, $t_b$. Those branch-specific rates are modulated by family-specific rate factors, which determine the evolutionary rates of families relative to each other. Domain family evolution will progress faster on some branches than on others, depending on the branch-specific rates. On any given branch, however, families with lower family-specific rates will evolve more slowly than families with higher family-specific rates.

The COUNT inference process proceeds in two passes. In the first pass, model parameters are estimated by likelihood maximization. In the second pass, the parameter values estimated in Pass 1 are used to calculate the expected values of ancestral quantities of interest.

*PASS 1:* In COUNT, parameter estimation is implemented as an iterative numerical maximization procedure (Csűrös and Miklós 2009). Parameter estimation is considered to have reached convergence when the increase in the log-likelihood between consecutive iterations is less than 0.01. This inference process estimates the Poisson parameter ($\phi$) and the branch-specific parameters ($t_b,\lambda _b, \kappa _b, \forall b \in B$). The optimization process also fits the discretized gamma distributions for family-specific variation.

We applied the COUNT model to the species tree in Fig. A1 and a table of 1393 phylogenetic profiles, described in Data above. The maximum likelihood parameter estimation procedure was carried out in stages of increasing model complexity to facilitate convergence, as recommended in the COUNT manual (Csűrös 2009). In the first stage, the values of $\sigma , \kappa , \lambda , \mu$ and $\phi$ are estimated with no rate variation. These serve as starting estimates for the next stage, which models branch-specific variation, only. The resulting branch-specific parameter values are used as starting estimates for the third stage, which comprises both branch- and family-specific variation. The inferred parameter values obtained in the final stage are given in Table A4 and Fig. A2.

*PASS 2:* The results of this maximum likelihood inference procedure carried out in the first pass are used to estimate three quantities: family-specific rate parameters, the expected domain repertoire in each ancestral genome, and the changes in each family on each branch of the species tree.

*Family-specific rate factors* The first pass infers the midpoints of the Slow and Fast rate categories, $\pi ^S$ and $\pi ^F$, for each parameter $\pi \in \{\sigma _f, \kappa _f, \lambda _f, \mu _f\}$. In the second pass, COUNT determines the probabilities that family *f* is evolving at the slow rate ($p_f(\pi ^S)$) or the fast rate ($p_f(\pi ^F)$). From these estimated probabilities, we calculated $\pi _f = \sum _{c \in \{S,F\}} p_f(\pi ^c) \pi ^c$, the expected value of $\pi$ for each family. The distributions of these family-rates are shown in Fig. A3. Scaled family-specific rates, $\sigma _f \lambda _f$, $\sigma _f\kappa _f$, and $\sigma _f \mu _f$, were also calculated for each domain family (Fig. A4) for use in downstream analyses.

*The ancestral domain family repertoire* The expected number of families encoded in each ancestral genome is also determined in the second pass. The ancestral domain repertoire can further be partitioned into the expected numbers of singleton families and multicopy families. To reduce the number of parameters, COUNT only considers three states: the family is absent ($n=0$), present in a singlecopy ($n=1$), or present in more than one copy ($n>1$). For each ancestral node in $v \in V$, the probabilities $p_f(v, n=0)$, $p_f(v, n = 1)$, and $p_f (v, n > 1)$ are determined for every family *f*. From these probabilities, we calculated the expected numbers of families that are absent, singletons and multicopy in ancestor *v*, given by $\sum _f p_f(v,n=0)$, $\sum _f p_f(v,n=1)$ and $\sum _f p_f(v,n>1)$, respectively. In this study, the ancestral domain repertoire is represented as the expected numbers of singlecopy and multicopy families (and not the expected number of domains) encoded in an ancestral genome, because COUNT does not estimate the expected number of copies in a multicopy family. In present-day genomes, the numbers of domains from each family encoded in the genome are known. Although the actual present-day family sizes are known, in some figures we represent present-day genomes in terms of the fraction of singleton or multicopy families, to allow for direct comparison between the ancestral and present-day domain family repertoires.

*Ancestral domain family events* In Pass 2, COUNT outputs the probability of each possible state change (0 to 1, 1 to many, many to 1, 1 to 0) on every branch $b=(u,v)$ in the species tree. Specifically, COUNT considers the following *family events*: family *f* *originated* on *b* (i.e., *f* was not encoded in species *u* and was encoded in species *v*, where *u* is ancestral to *v*); *f* *expanded* on *b* (i.e., *f* was encoded in a singlecopy in *u* and more than one copy in *v*); *f* *contracted* on *b* (i.e., *f* was a multicopy family in *u* and singlecopy family in *v*), *f* *expired* on *b* (i.e., *f* was encoded in *u*, but not in *v*); or *f* remained unchanged. (Note that domain *family events* are not synonymous with the underlying *domain events*. For example, a family extinction is always the result of a domain loss, but a domain loss only results in a family extinction, when the family is size one.)

In the the second pass, for each domain family, *f*, and each branch, *b*, COUNT estimates the probability that each of these four domain family events occurred. From these probabilities, we estimate the expected number of family events of each type on each branch. For example, the expected number of families that originated on *b* is the sum of the probability, for each family *f*, that *f* originated on *b*. The expected numbers of families that expanded, contracted or expired on *b* are calculated analogously.

 In summary, we used the phylogenetic profiles of 1393 domain families across 24 species as input for the Birth-Death-Gain (BDG) implemented in COUNT. COUNT’s inference process operates in two passes, as described above.

In Pass 1, we obtained maximum likelihood estimates for several key parameters: the Poisson parameter at the root, the branch-specific parameters ($t_b,\lambda _b, \kappa _b$, for all $b \in B$), and the family-specific rate category parameters. The latter include the gamma shape parameter and the midpoints of the fast and slow rate categories for all family-specific parameters $\pi \in \{\sigma _f, \kappa _f, \lambda _f, \mu _f\}$. These estimates are summarized in Table A4. The family-specific gamma distributions are presented in Fig. A2.

Using prior probabilities derived from the parameter estimates obtained in Pass 1, Pass 2 yielded expected family-specific rate factors, shown in Fig. A3. From these, we calculated scaled family-specific rates ($\sigma _f \lambda _f$, $\sigma _f\kappa _f$, and $\sigma _f\mu _f$) for each domain family for use in downstream analyses, as reported in Fig. A4.

In addition, Pass 2’s results enabled the calculation of the expected number of singlecopy and multicopy families in each ancestral genome, as well as the expected number of families that originated, expanded, contracted, and went extinct on each branch for downstream analyses. These calculated data are documented in Supplementary Note 1.

After completion of Pass 2, we removed 88 domain families that are present only in the three outgroup species (Fig. A1a), which were included solely for COUNT’s inference process. Moreover, in Pass 2, COUNT failed to return estimates for 22 large families (see Table A3). These families were also excluded from downstream analyses. These filtering steps yielded a final set of 1283 domain families used in downstream analyses.

### Dollo Parsimony Inference

Many prior studies of the evolution of the metazoan protein repertoire have used Dollo parsimony for ancestral inference. In order to compare our results to those of prior work,we also performed ancestral reconstruction using Dollo parsimony. The Dollo parsimony method takes a rooted species tree and a set of phylogenetic presence/absence profiles as input and infers the presence or absence of each family in each ancestral node, under the assumption that a domain family can be gained only once, but can be lost multiple times. We applied the Dollo parsimony implementation in COUNT to presence-absence profiles of the 1371 domain families in 24 species,using the full species tree in Fig. A1.

### Functional Annotation of Domain Families

For functional analyses in this study, we use the domain-specific functional ontology developed by Vogel et al. (2004, 2005), which is hosted by the SUPERFAMILY database (https://supfam.org/SUPERFAMILY/function.html). The Vogel ontology is, to our knowledge, the most comprehensive functional annotation available that is specifically designed for protein domains. The “InterPro2GO” project provides manual annotation of domains with GO terms, based on experimental characterizations of the domain’s function (Burge et al. 2012; Blum et al. 2025). However, at the time of writing, only 30% (395) of the 1283 families in this study have been assigned one or more GO terms. In contrast, 1199 domain families are annotated in the Vogel ontology (Table A7).

**Fig. 3:**
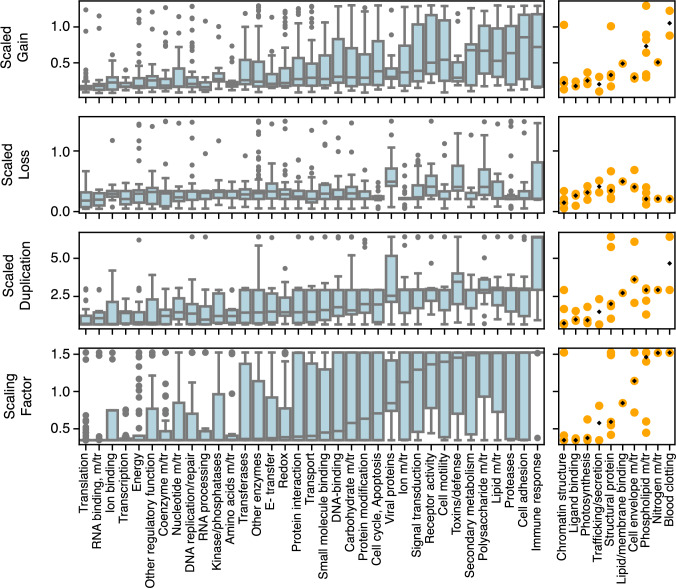
Association of family-specific rates and functional categories: Scaling factor ($\sigma _f$) and scaled duplication ($\sigma _f\lambda _f$), loss ($\sigma _f\mu _f$), and gain ($\sigma _f\kappa _f$) rate distributions in 45 functional categories (Vogel et al. 2004, 2005). Categories are ordered by ascending median scaling factor value. Left: Box-and-whisker plots of rate distributions in categories with 9 or more domain families. Right: Rates in categories with fewer than 9 families are plotted as individual points in yellow; medians are shown as black points. Abbreviation: m/tr = metabolism and transport

The Vogel ontology consists of 49 detailed functions grouped into 7 general functions. The ontology is constructed such that each domain is mapped to at most one general and one detailed functional category. Two detailed functions are not represented among the domains in our data set. In addition, two detailed functions, “general” and “unknown function”, are not informative. The 127 domains assigned to these uninformative categories were excluded from functional analyses (Fig. 3 and Tables 1, A9), leaving 1072 domain families in 45 detailed categories.

Among the 45 categories, 35 categories contain at least 9 domain families; these categories comprise 1041 domains, in total. The medians of the scaling factors ($\sigma _f$) within these categories are bimodally distributed. To rule out the possibility that this bimodal distribution is an artifact of the underlying distribution of $\sigma _f$ itself (Fig. A3), we performed a randomization procedure with the difference between the largest and smallest median as a test statistic. The association between families and functional categories was permuted, randomizing the scaling factors assigned to each category, and the median scaling factor was calculated for each category (Fig. A5). In 1000 permutations, the difference between the maximum and minimum medians was smaller in the permuted data than in the genuine data in every replicate, indicating a significant deviation from expectation ($p=0$).

### Clustering of Event Rates

We applied Statistical Hierarchical Clustering (SHC) to the 1283 family-specific scaled rate profiles ($\sigma _f \lambda _f$, $\sigma _f\kappa _f$, and $\sigma _f \mu _f$) to determine whether groups of domains share distinct rate profiles. SHC (Kimes et al. 2017) extends traditional hierarchical clustering by providing a statistical assessment for the clustering results obtained. Hierarchical clustering is the process of repeatedly merging clusters of points, generating progressively larger clusters until a termination criterion is satisfied. At each iteration, the pair of clusters to be merged is selected according to a protocol that depends on the inter-cluster distances between all pairs. Inter-cluster distances are recalculated and the process repeats. Hierarchical clustering methods differ in the metric used to calculate pairwise distances, the protocol for selecting a pair to merge, and the termination criterion.

SHC offers a choice of metrics and merging protocols and a termination criterion that ensures that the resulting clustering deviates significantly from a null model. At every merge point in the agglomerative procedure, SHC calculates a test statistic that compares pairwise distances within each cluster, considered separately, with pairwise distances in the set resulting from their union. The statistic under the null hypothesis is estimated by Monte Carlo sampling from a Gaussian distribution derived from the clusters to be merged. The *p* value is the fraction of replicates that had a better statistic than the genuine bipartition. The SHC termination condition ensures that the significance of each cluster in the final clustering exceeds a user-provided significance threshold, $\alpha$, after family-wise error correction. SHC guarantees $p < \alpha$ for every cluster in the clustering; in some cases, the *p* values associated with the individual clusters are much smaller than $\alpha$. We report the largest (i.e., least significant) *p* value for each clustering.

The scaled rates were clustered with four metrics (Euclidean, Pearson, Manhattan, Maximum) and five agglomeration strategies (Average, Median, Complete, Centroid, Single) available in the SHC package. Prior to clustering with SHC, the data was centered and scaled using the R Scales package (Wickham et al. 2025). Domain families with identical rate profiles were removed, reducing the input by 64 families.

For downstream analyses, the excluded domain families were restored by assigning each family to the same cluster as their corresponding identical rate profile. Of these 20 combinations, 10 yielded significant clusterings at the $\alpha = 0.05$ level (Table A1). At this level of significance, the chance expectation of a significant clustering is one in 20. All clusterings have a mean silhouette (Rousseeuw 1987; Maechler et al. 2023) above 0.5, suggesting that the clusterings are reasonably coherent.

We next asked whether the 10 significant clusterings capture similar relationships between domain families, using Normalized Mutual Information (NMI), an information-theoretic measure that quantifies the amount of shared information between two distributions. The raw mutual information is normalized by the clustering entropy, providing a scale-independent measure that ranges from zero to one. NMI is widely used in computational biology due to its interpretability and sensitivity to clustering granularity (Vinh et al. 2010; Strehl and Ghosh 2002; Cover 1999; Rodriguez et al. 2019; Chiquet et al. 2020). The NMI values for our clusterings suggest that, with the exception of clusterings that used average linkage, there is substantial overlap in the information they encode (Fig. A6). The Euclidean-Centroid clustering was selected for further analysis.

## Results

We investigate the evolution of the protein toolkit in 21 genomes, comprising two choanoflagellate genomes and 19 metazoan genomes, ranging from sponge to human. These genomes encode 1283 domain families, as defined by the SUPERFAMILY database; 501 core families are found in all 21 genomes and 547 are common to Metazoa. Among the non-core families, 84 are species-specific (Table A5). Notably, 37 of these are found in sponge. The present-day phylogenetic profiles of non-core families show additional taxonomic structure (Fig. A7), including families found uniquely in choanoflagellate, metazoan, and vertebrate genomes. Idiosyncratic absences are also observed. A substantial number of families that are broadly represented in metazoan genomes are absent from the lamprey genome, and to a lesser extent from the sea squirt, nematode, tick, and fruitfly genomes.

### Family-Specific Domain Rate Variation

We used the Birth-Death-Gain model implemented in COUNT (Csűrös 2010) to infer the family-specific rate factors that maximize the likelihood of present-day holozoan domain family sizes. For each family, *f*, among the 1283 distinct domain families in the 21 species analyzed, this yielded family-specific gain, duplication, and loss rates ($\lambda _f, \mu _f$, and $\kappa _f$), as well as a scaling factor ($\sigma _f$) that captures the rate of change of the family, overall.

To examine the relationship between domain rates and domain function, we used a manually curated, domain-specific ontology (Vogel et al. 2004, 2005), consisting of 49 detailed functions grouped into 7 general functions (Table A7). Under this ontology each domain is mapped to at most one general and one detailed functional category. Domain families lacking an informative annotation (see Methods) were excluded from functional analyses, resulting in 1283 families distributed across 45 rate categories.

Figure 3 shows the distribution of the overall scaling factor ($\sigma _f$) and the scaled duplication ($\sigma _f \lambda _f$), gain ($\sigma _f \kappa _f$), and loss ($\sigma _f \mu _f$) rates in each functional category represented in our data set. Functional categories are ordered according to the median scaling factor ($\sigma _f$, bottom panel) in each category. The distribution of the median scaling factor across functional categories is bimodal. In 15 of 35 categories that contain at least 9 domain families (box-and-whisker plots in Fig. 3), the median is within 10% of the minimum scaling factor. At the other end of the scale, in 8 categories the median is within 10% of the maximum scaling factor. In other words, in 23 out of 35 rate categories, at least half of families are within 10% of an extreme value in the scaling factor distribution. This distribution of rates across functional categories deviates significantly from chance expectation (*p* < 0.001, permutation test, see Methods). This supports the hypothesis that domains with similar functions also have similar rates.

We further observe that more than half of the functional categories are preferentially associated with either very low rates or very high rates. The fifteen functional categories with a preponderance of low scaling factors are associated with basic cellular processes found in all cells across the tree of life: translation; RNA binding, metabolism/transport; ion binding; transcription; energy; other regulatory function; coenzyme metabolism/transport; nucleotide metabolism/transport; DNA replication/repair; RNA processing; kinases/phosphatases; amino acids metabolism/transport; transferases; other enzymes; and E- transfer. The eight functional categories with functions within 10% of the maximum scaling factor (cell motility; toxins/defense; secondary metabolism; polysaccharide metabolism/transport; lipid metabolism/transport; proteases; cell adhesion; and immune response) are associated with metazoan innovations, including multicellularity, tissue repair, and innate and adaptive immune response.

### The Evolution of the Ancestral Domain Repertoire

We next probed the evolutionary history that gave rise to the present-day metazoan domain family repertoire. The fraction of the 1283 families encoded in present-day genomes ranges from 66% in the choanoflagellate *M. brevicollis* to 81% in human; 41% to 61% of these families, respectively, are present in more than one copy. Within this range, protein domain content varies considerably. For example, the domain repertoire in Nematostella is 7% larger (85 families) than the repertoire of Hydra, its closest relative.

**Fig. 4:**
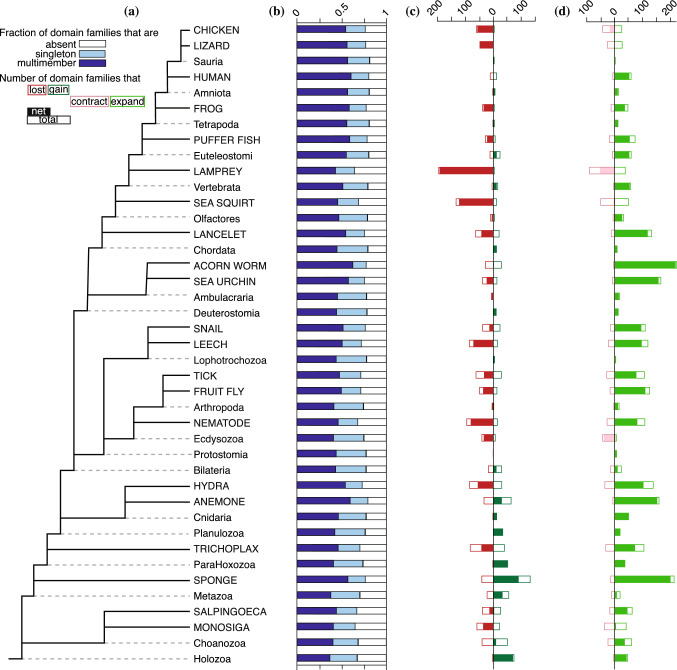
**a** Phylogeny of the holozoan genomes used in this study. Names in all capitals indicate present-day species; names in title case indicate ancestral taxa. Solid lines indicate species tree branches. Dashed lines connect the names of ancestral taxa to the corresponding internal nodes in the tree. The full tree with outgroups is shown in Fig. A1a; the root of the tree shown here corresponds to the Opisthokonta-Holozoa branch in the full tree. **b** The expected fraction of the 1283 domain families that are absent (white), singlecopy (light blue), and multicopy families (dark blue) in present-day and ancestral species. **c** The expected numbers of families that originated (dark green) and went extinct (red), open rectangles. Net change, solid rectangles. **d** The expected numbers of families that expanded (green) and contracted (pink), open rectangles. Net change, solid rectangles

To better understand the history of this domain repertoire variation, we used COUNT to estimate the expected number of families that were present in each ancestral genome (Fig. 4b). According to this reconstruction, approximately 68% of the 1283 present-day domain families were encoded in the holozoan ancestor, increasing to 78% in the bilaterian ancestor and 81% in ancestral genomes in the vertebrate clade. Of the families present in these ancestral genomes, the percentage of multicopy families ranges from 55% in Holozoa to 70% Amniota.

This reconstruction provides an estimate of the size of the protein domain repertoire encoded in ancestral genomes, but does not reveal to what extent the specific families that made up these repertoires changed over the course of metazoan evolution. Comparing the size of the expected repertoires of the ancestor and descendant in a given species tree lineage gives the *net* change in the numbers of families encoded. To assess the plasticity of the ancestral repertoire, we calculated the expected number of family gains and losses on each branch of the species tree (Fig. 4c) and compared the number of families that sustained a change to the net change in expected repertoire size.

**Fig. 5:**
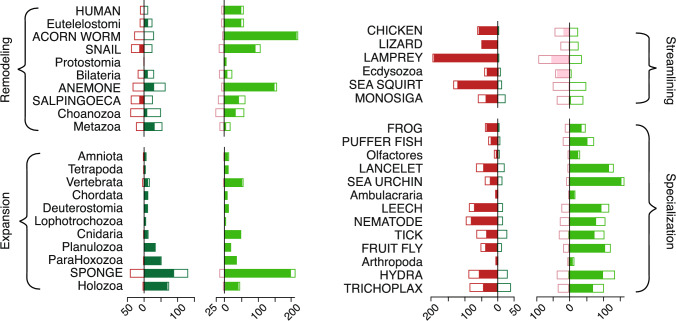
Evolution of the protein domain repertoire characterized by four evolutionary modes: *Expansion*, lineages in which expected family gains exceed expected family losses by a factor of two or more and the expected number of family expansions is at least twice that of family contractions. *Remodeling*, lineages in which the net change in the number of families encoded in the genome is less than 60% of the sum of families gained and lost and the number of expansions is greater than the number of contractions. *Specialization*, lineages in which losses exceed gains by a factor of two and the expected number of expansions is at least twice the number of contractions. *Streamlining*, lineages in which family losses exceed gains by a factor of two and family expansions do not exceed the family contractions by more than 20%. With the exception of Monosiga and Lizard, more families are contracting than expanding in these genomes. Legend as in Fig. 4

In some genomes (Fig. 5), the expected gains and losses are well aligned with the difference in repertoire sizes. For example, the Planulozoan repertoire encoded 984 families, 33 more than ParaHoxozoa, its immediate ancestor. Consistent with this, 33 family gains and no family losses were inferred in this lineage.

In other lineages, however, the inferred family events reveal much more activity than is apparent from the change in the ancestral domain repertoire. For example, the Bilaterian ancestor encoded 12 more families than its immediate ancestor (996 versus 984); yet the expected number of family gains is 29, which were offset by 18 expected losses. Here the number of families gained and lost greatly exceed change in the size of the repertoires. This indicates *turnover* in the composition of the domain repertoire: continual family loss and replacement, resulting in substantial change in functional capabilities. A similar pattern of family turnover, with substantial actual change but little net change, is seen in the Metazoan, Choanozoan and Euteleost lineages, as well as in several terminal branches.

Having observed substantial plasticity in protein domain repertoire composition, we wondered whether there is similar plasticity in domain family size. To probe this question, we calculated the expected numbers of family expansions and contractions on each branch (Fig. 4d) and compared that with the net change in the size of the multicopy domain repertoire.

A number of lineages, however, exhibit substantial turnover in the specific families that make up the multicopy repertoire. For example, the estimated increase in the number of multicopy families in the Bilaterian genome is 9. However, the expected number of family expansions is 23, offset by 14 expected family contractions. The number of families that changed in size is four-fold greater than the increase in the number of multicopy families. Thus, Bilateria exhibits substantial turnover in family size, as well as in family presence.

These examples reveal distinct modes of domain repertoire evolution (Fig. 5). A pattern of steady growth exhibits repertoire *expansion*, in which both the number and the size of domain families increases. This is observed in 11 of the 40 lineages (contemporary and ancestral) in our study. In contrast, our reconstruction suggests substantial *remodeling* of the structural and functional capabilities of other genomes, such as the Bilaterian repertoire. 9 other genomes also exhibit this pattern.

The remaining genomes are characterized by a net decrease in the size of the domain repertoire (Fig. 5, right). In 13 genomes, (e.g., lancelet and sea urchin), this is accompanied by an increase in the number of multicopy families. This suggests a process of repertoire *specialization*. The variety of functional and structural modules decreases, but those families that are retained increase in size. The remaining 6 genomes manifest *streamlining* of the domain repertoire, in which the number of domain families decreases and family size either decreases or remains roughly constant.

### Are Groups of Domains Evolving in Concert?

The emergence of phenotypic novelty is associated with expansion and elaboration of existing cellular pathways and protein complexes, as well as the appearance of new ones (Aziz and Caetano-Anollés 2021; Babonis and Martindale 2017; Bornberg-Bauer et al. 2005; Patthy 2021; Pires-daSilva and Sommer 2003). This hypothesis predicts that domains that mediate protein-protein interactions in the same cellular pathways and proteins complexes will tend to have similar rate profiles. In addition, the frequency of domain pairs (domain bigrams) in multidomain sequences deviates significantly from expectation, given domain unigram frequencies (Cui et al. 2024, 2022; Vogel et al. 2005), suggesting that the formation of domain combinations is selectively constrained. Under these constraints, domains that frequently co-occur will also tend to have similar duplication, gain, and loss rates.

With this in mind, we asked whether there are groups of domains that exhibit greater similarity in rate profiles than expected by chance. To identify such groups, if they exist, we applied statistical hierarchical clustering (Kimes et al. 2017) to the set of 1283 family-specific scaled rate profiles $\{\sigma_f\lambda _f, \sigma _f\mu_f, \sigma_f \kappa _f\}$, using five agglomeration strategies and four metrics, as described in Methods. Ten of these 20 combinations yielded significant clusterings at the $\alpha = 0.05$ level (Table A1). Since less than one clustering in 20 is expected by chance at this significance level, this is strong evidence that groups of domains share evolutionary trajectories.

**Fig. 6:**
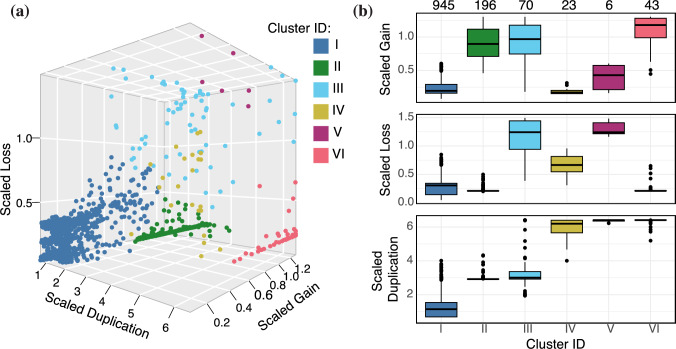
Domain family-specific rates for scaled gain ($\sigma _f\kappa _f$), loss ($\sigma _f\mu _f$), and duplication ($\sigma _f\lambda _f$): **a** Scaled family rates, where each point is a domain family that is colored according to the cluster assigned by statistical hierarchical clustering (Kimes et al. 2017) with centroid linkage and the Euclidean metric. **b** Box-and-whisker plots of the distributions of family rates, by cluster. Boxes are colored according to clustering results and ordered by median duplication rate. Upper horizontal axis provides the number of domains in each cluster

We selected the Euclidean-Centroid clustering for further analysis, because it is highly significant ($p < 3\textrm{e}{-17}$) and is representative of the majority of clusterings obtained (Fig. A6). Each cluster in this grouping has a characteristic rate profile (Fig. 6). Cluster I, by far the largest cluster, has slow gain, duplication, and loss rates. Clusters II and II, which are intermediate in size, have moderate duplication rates and high gain rates, but differ in their loss rates. The three smallest clusters (IV–VI) are characterized by very high duplication rates.

**Table 1:** Distribution of general functions (Vogel et al. 2005) across scaled family rate clusters

General function	I		II		III		IV–VI		Total	
Extra-cellular	**16**	**2%**	13	7%	4	7%	**13**	**22%**	46	4%
General	55	7%	12	7%	5	8%	4	7%	76	7%
Information	**157**	**20%**	**10**	**5%**	3	5%	**1**	**2%**	171	16%
Intra-cellular	**99**	**13%**	**54**	**30%**	8	13%	14	24%	175	16%
Metabolism	324	42%	57	31%	31	52%	13	22%	425	40%
Regulation	110	14%	36	20%	7	12%	9	16%	162	15%
Viral	11	1%	0	0%	2	3%	4	7%	17	2%
Total	772		182		60		58		1072	

“Viral” indicates families assigned to the only informative functional annotation in the “Other” general category. Bold indicates domain family counts that deviate from chance expectation ($p < 0.05$ with multiple testing correction; hypergeometric test). *p* values are given in Table A9

In order to better interpret these differentiated rate profiles, we examined the functional profile (see Methods) for each cluster (Tables 1 and A8). Since Cluster IV–VI are very small and share a very high duplication rate, we combined them for the purposes of functional enrichment analysis. After multiple testing correction, we find that Cluster I is significantly enriched for domains with information processing functions (hypergeometric test, $p<0.05$). Domains with roles in extra- and intra-cellular processes are underrepresented. In contrast, in Cluster II, information processing is underrepresented and intra-cellular processes are over represented. Taken together, domain families with extra-cellular functions are enriched in the three clusters with very high duplications. Only one domain with an information processing function is found in these three clusters, combined.

**Fig. 7:**
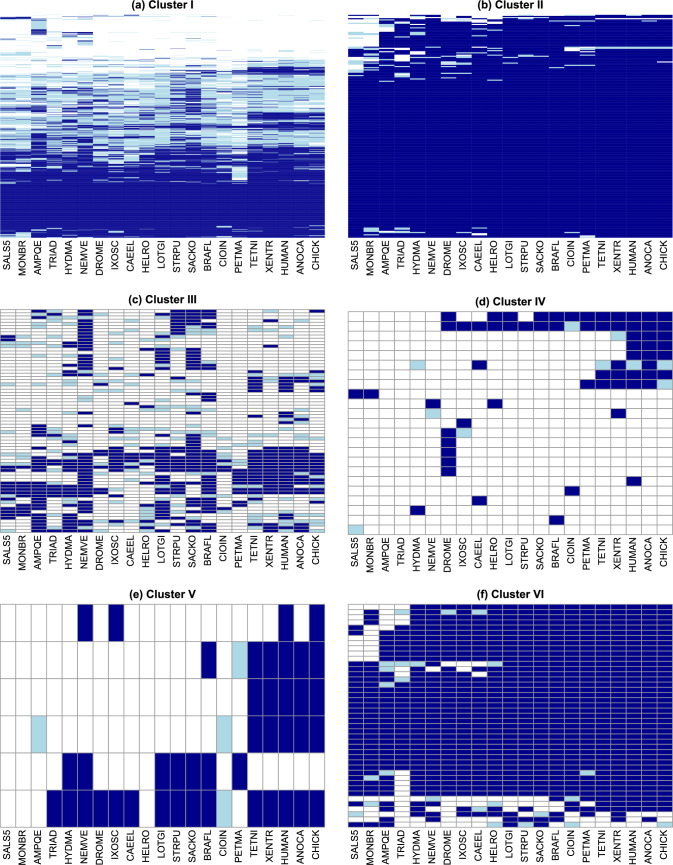
Distribution of domain families in each cluster over 21 genomes in present-day species. Rows represent domain families; columns represent present-day species. Cells are colored according to whether the domain was absent (white) or presence as a singleton (light blue) or multicopy (dark blue) family in that species. In each subfigure, domain families were ordered according to profile similarity by the *pheatmap* package in R (Kolde 2018). Species names abbreviated as follows: SALS5 (SALPINGOECA), MONBR (MONOSIGA), AMPQE (SPONGE), TRIAD (TRICHOPLAX), NEMVE (ANEMONE), HYDMA (HYDRA), CAEEL (NEMATODE), DROME (FRUIT FLY), IXOSC (TICK), HELRO (LEECH), LOTGI (SNAIL), STRPU (SEA URCHIN), SACKO (ACORN WORM), BRAFL (LANCELET), CIOIN (SEA SQUIRT), PETMA (LAMPREY), TETNI (PUFFER FISH), XENTR (FROG), ANOCA (LIZARD), CHICK (CHICKEN)

Next, we asked how the domains in each cluster are distributed across present-day genomes (Fig. 7). About a third of the 945 domains in Cluster I are present in all 21 genomes in this data set. Another 10% are present in a single species, consistent with lineage specific gains. The remaining families in Cluster I have a patchy distribution. Perhaps a quarter of domain families in this cluster are present in one or a small number of genomes only. This includes the 37 sponge-specific domain families, for example. Clusters II and VI are primarily made up of core families (Table A6). Almost 75% of Cluster II families and 40% of Cluster VI families are encoded in all present-day holozoan genomes in our study. Eighty percent of Cluster VI families are in the Bilaterian core. The vast majority of domain families in both clusters are multicopy. In contrast, the taxonomic distribution of Cluster III is patchy: 68 of 70 families are encoded in at least two, but not all, genomes. Cluster V is similarly patchy. Cluster IV families also have sparse phylogenetic profiles, but with less patchiness: half of Cluster IV families are present in only one species and another quarter are specific to a single taxonomic clade.

**Fig. 8:**
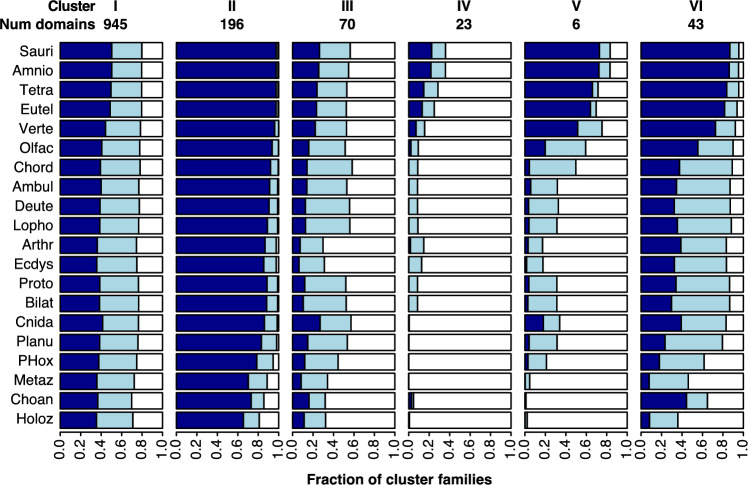
Ancestral family compositions in Clusters I–VI. The expected frequency of absent (white), singleton (light blue), and multicopy families (dark blue) in ancestral species in each cluster. Expected family counts are normalized by cluster size. Species are listed in the same order as the ancestral nodes in Fig. 4. Taxa abbreviated as follows: Sauri (Sauria), Amnio (Amniota), Tetra (Tetrapoda), Eutel (Euteleostomi), Verte (Vertebrata), Olfac (Olfactores), Chord (Chordata), Ambul (Ambulacraria), Deute (Deuterostomia), Lopho (Lophotrochozoa), Arthr (Arthropoda), Ecdys (Ecdysozoa), Proto (Protostomia), Bilat (Bilateria), Cnida (Cnidaria), Planu (Planulozoa), Metaz (Metazoa), Choan (Choanozoa), Holoz (Holozoa)

**Fig. 9:**
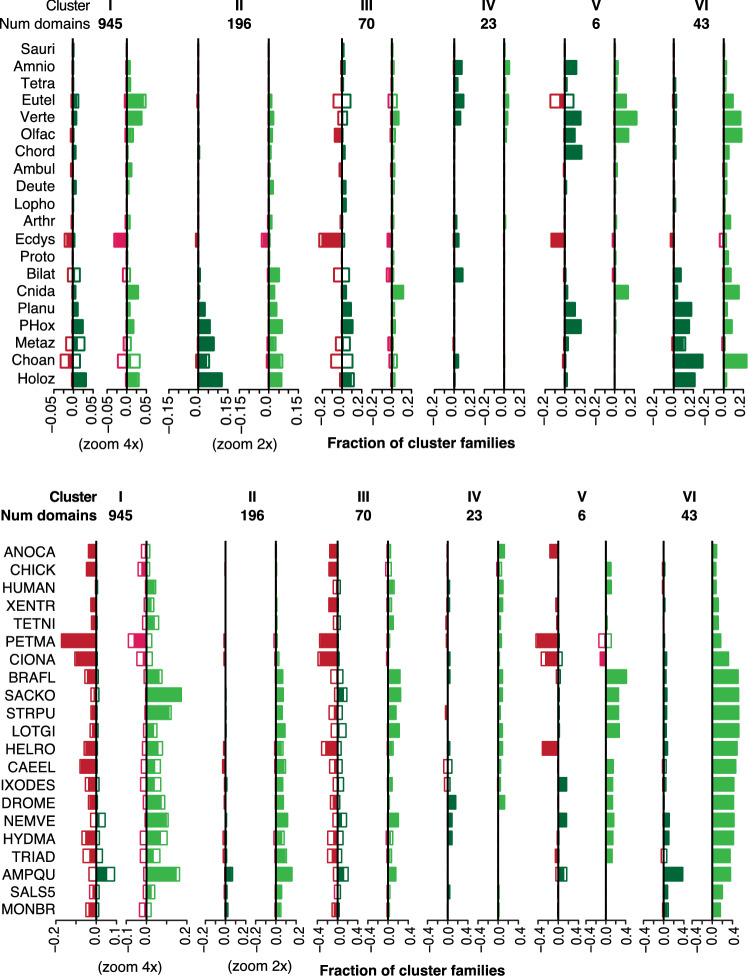
Expected family events in Clusters I–VI. For each cluster, the expected fraction of cluster families that originated (dark green) or went extinct (red) and, expanded (light green) or contracted (pink) in ancestral (top panel) and terminal (bottom panel) lineages. Expected family events are normalized by cluster size. Species listed in the same order as in Fig. 4; names abbreviated as in Figs. 7 and 8. Open and filled rectangles defined as in Fig. 4

The taxonomic distributions of individual clusters range from very dense to patchy to very sparse. The patchy distributions must be the result of parallel gains, parallel losses or reversals. Clusters with very dense or very sparse clusters can be explained with minimal parallelism, but might also conceal a higher degree of homoplasy. To better understand the evolutionary processes that gave rise to these present-day distributions, we examined the ancestral states (Fig. 8) and the pattern of family gain, expansion, contraction, and loss associated with the families in each clusters (Fig. 9).

Cluster I families display a mix of core, patchy, and sparse phylogenetic profiles. This mix is consistent with the relatively broad event rate distributions in Cluster I. The median gain, duplication, and loss rates are low, but the upper quantiles in all three distributions are in the mid-range. In the reconstruction, each ancestral genome encodes an expected 75–80% of Cluster I families, with multicopy families ranging from 40% in the holozoan ancestor to 50% in euteleosts. The frequencies of singleton and multicopy Cluster I families encoded in ancestral genomes are remarkably stable. However, the expected number of family gain and family loss events are consistent with ongoing family turnover (Fig. 9), which suggests that the actual families vary from ancestor to ancestor.

The histories of family events in Clusters I and III show family turnover in the choanozoan, metazoan and bilaterian ancestors. Similarly, turnover is evident in the euteleost ancestor in Clusters III and V. Many terminal lineages also indicate substantial turnover, especially in Cluster III. In Cluster I, family events on terminal branches indicate a mixture of turnover and specialization. In all three clusters, the present-day patchy distributions, combined with ongoing loss, replacement and resizing of families, is suggestive of extensive remodeling of the protein domain repertoire.

In contrast, very little turnover was inferred for Cluster IV families. The present-day profiles of this cluster are so sparse that this cluster can be explained by a small number of gains.

It is instructive to compare the evolutionary histories of Clusters II and VI, which have similar, dense taxonomic distributions. The reconstruction of domain families encoded in ancestral genomes suggests that roughly 80% of Cluster II families were already present in the holozoan ancestor; virtually all families were encoded in the planulozoan ancestor. In all ancestral nodes, the majority of families in the expected repertoire are encoded in two or more copies. The expected family events in ancestral lineages consist of family gains and expansions (Fig. 9). In summary, Cluster II exhibits a consistent, gradual expansion of a stable metazoan protein domain toolbox.

In contrast to Cluster II, the acquisition of the full complement of Cluster VI multicopy families occurred more recently than their common ancestor. Although an expected 85% of families were encoded in the Bilaterian ancestor, most of these were singletons; increases in copy number occurred much later in the Chordate clade. The distribution of inferred family gains across ancestral species is similar to that of Cluster II. However, family expansion continued in recent ancestors and in many invertebrate terminal lineages (Fig. 9), consistent with the higher duplication rates in Cluster VI. Note that this inferred history differs from a parsimony-based ancestral reconstruction, which would predict a full suite of multicopy families in the Bilaterian ancestor.

**Fig. 10:**
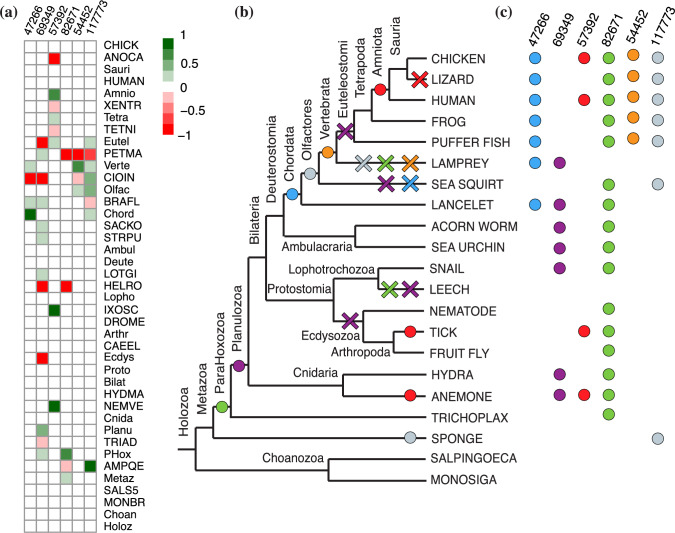
Evolutionary history for the six domain families in Cluster V, based on event probabilities. **a** Heatmap of the probability of family gain minus loss on each species tree branch. Superfamily IDs are listed on top. Species listed in the same order as in Fig. 4; names abbreviated as in Figs. 7 and 8. **b** High-confidence family gain (filled circle) and loss (cross) events mapped to the species tree. High-confidence events are defined as those with probability greater than 0.6. Colors correspond to domain family as displayed in (**c**). **c** Family presence in the genomes of present-day species, indicated by filled circles. Each domain family is assigned a distinct color.

### Parallel Gains and Losses

The present-day phylogenetic distribution (Fig. 7) reveals a number of families that are present in distantly related taxa, but absent in their closest relatives, which suggests a history of independent gains, independent losses, and reversals. These patchy distributions are particularly evident in clusters with high loss and/or gain rates.

To determine whether individual families are sustaining parallel gains and parallel losses, for each family, we examined the probability of a family gain or loss event on each branch of the species tree (Figs. A8–A11). In this case, we can not rely on the expected number of events across all domain families, but focus on the probability of an event in each individual family. This is accomplished by applying a threshold to focus on cases where the probability of origination is much higher than that of other events. We say that family *f* has sustained a high-confidence gain event in species *v* if the difference between the gain and loss probabilities of *f* is greater than 0.6. Similarly, we say *f* was lost on *b* if this difference is less than $-0.6$. This analysis identified 115 families that have a history of at least two high-confidence gain events. Many of these family sustained multiple independent gains, especially in Clusters III and VI which have high gain rates. Taken together, these 115 families comprise 315 parallel gains. Many instances of families with a high probability of extinction in distinct lineages were also identified: 300 families exhibited a history of two or more high-confidence loss events, representing 1085 parallel family loss events, in total.

These trends are exemplified by the six families in Cluster V (Fig. 10). In the BDG reconstruction, three of the six families (47266, 69349, 82671) have parsimonious histories with a single gain event and are, therefore, also consistent with the Dollo parsimony criterion. Two more families (57392, 117773) have histories with multiple, independent gains. Note that for both families, a much larger number of events would be required for a history that satisfies the Dollo parsimony criterion. The BDG reconstruction for the remaining family (54452) does not satisfy any parsimony criterion: this family is gained in the vertebrate ancestor, but is immediately lost in lamprey. A history comprising a gain in the euteleost ancestor requires fewer events.

**Fig. 11:**
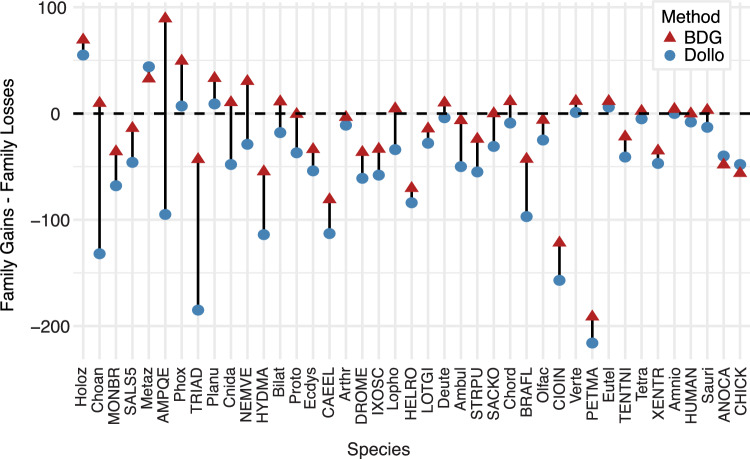
Comparison of BDG and Dollo estimates for net change in expected family representation on each species tree lineage. Expected family gains minus expected family losses, as estimated by the Birth-Death-Gain model (triangles), are compared to gains minus losses estimated by Dollo parsimony (circles). Vertical lines indicate the differences between each estimation. Species listed in the same order as in Fig. 4; names abbreviated as in Figs. 7 and 8

The prevalence of parallel gains inferred by the BDG model led us to wonder how Dollo parsimony, which is widely used for investigating the evolution of the protein-coding repertoire (e.g., Zmasek and Godzik 2011; Fernández and Gabaldón 2020; Guijarro-Clarke et al. 2020; Domazet-Lošo et al. 2024; Fairclough et al. 2013; Qiu et al. 2015, 2018; Bowles et al. 2020), would perform on this data set. According to the Dollo parsimony criterion, a family can be gained only once, but can be lost multiple times. Unlike the BDG method, which allows for parallel events of either type, under the Dollo criterion, a patchy distribution can only be explained by an early gain, followed by multiple independent losses. To investigate the impact of this constraint, we applied the Dollo parsimony reconstruction method implemented in COUNT (Csűrös 2010) to infer the number of families gained and lost on each branch, using the same input data as for the BDG model. We examined the net change (gains minus losses) estimated by both models on each branch (Fig. 11). The results show that Dollo parsimony has an overwhelming preference for family losses, compared with the BDG model: In the Dollo reconstruction, family losses exceeded gains on 13 of 20 internal branches in the species tree; in the BDG reconstruction, losses dominated gains on only 5 of 20 branches. Considering all branches combined, Dollo inferred a total of 300 gain events and 2239 loss events, while the BDG model inferred 895 expected family gains and 1477 expected losses.

## Discussion

In this work, we investigated the evolution of the protein domain repertoire in 21 holozoan species using the Birth-Death-Gain model implemented in COUNT (Csűrös 2010; Csűrös and Miklós 2009). We used the full COUNT model, which includes both branch- and family-specific rate variation. Incorporating family-specific variation has been shown to improve model fit, yielding more accurate estimators without sacrificing generality (Stolzer et al. 2014). Importantly, family-specific rates not only improve accuracy, but also provide insight into the evolutionary trajectories of individual domain families.

Using COUNT, we inferred the rates of domain gain, duplication and loss. When domain event rates are examined in light of functional properties, our results reveal that domains associated with basic cellular processes (e.g., transcription, translation, DNA replication, metabolism) tend to have slower evolutionary rates. In contrast, domain families with functions related to innovations that appear during metazoan evolution (e.g., cell adhesion, immune response, and cell motility) exhibit elevated rates.

We further asked whether there are cohorts of domains that are evolving in concert. We posit that if major events in organismal evolution are linked to changes in protein-mediated cellular processes, then domains associated with the requisite pathways and protein complexes will have similar rate profiles. Indeed, statistical hierarchical clustering (Kimes et al. 2017) of domain family rates revealed clusters that exhibit statistically robust rate differentiation. These clusters also share functional and historical attributes, consistent with a link between protein innovation and the emergence of phenotypic novelties.

Using the same model, we estimated the family sizes in ancestral genomes, as well as the expected family gains, expansions, contractions and extinctions in each lineage. We observed four distinct evolutionary modes that shape the protein domain repertoire in Metazoa. A small number of lineages are characterized by genome streamlining, that is, net loss of domain families and reduction in size among those that remain. Specialization of the protein domain repertoire, where the loss of some families is offset by a size increase in others, is a much more common evolutionary mode. A third set of lineages is characterized by gradual growth and expansion. Finally, and most compelling, we observe lineages where family turnover dominates. Notably, this process of remodeling, where some families are lost and are replaced by others, was inferred in the ancestors of several major clades, including Metazoa, Choanozoa, Bilateria and Protostomia, that are associated with dramatic and highly successful phenotypic innovations.

The limited importance of genome streamlining in this reconstruction stands in stark contrast to recent studies that report widespread loss in Metazoa (Guijarro-Clarke et al. 2020; Domazet-Lošo et al. 2024) and other multicellular groups (Qiu et al. 2018; Bowles et al. 2020; Zmasek and Godzik 2011). Many of these studies are based on analyses using Dollo parsimony, which, by design, resolves homoplasy with parallel losses. Reanalysis of our data set with Dollo parsimony dramatically increases the number of losses inferred. The ratio of losses to gains increases almost fivefold, from 1.7 losses for every gain obtained with the BDG model to 7.5 losses per gain as inferred by Dollo parsimony.

Our results demonstrate that, for this data set, the number of losses inferred is much greater with Dollo parsimony than with a Birth-Death-Gain model. While this discrepancy is noteworthy, the true history of the metazoan domain repertoire is unknown. We cannot rule out the possibility that Dollo parsimony is correctly inferring a history of massive loss. Nevertheless, there are several reasons to doubt that scenario. First, Dollo parsimony is designed to infer losses. Second, BDG models have the flexibility to model both parallel gains and parallel losses and, indeed, accounts of massive loss inferred with a BDG model have been reported (Csűrös and Miklós 2009). In a data set truly characterized by parallel loss, we might expect the two methods to produce similar results. Finally, a recent study of the evolution of the eukaryotic protein domain repertoire also reported that Dollo parsimony obtained much higher losses than a maximum likelihood method (Gàlvez-Morante et al. 2024). That study differed from ours in the taxonomic sample, the source of protein domain data, the implementation of Dollo parsimony and the specific probabilistic model used, emphasizing the potential role of Dollo parsimony in overestimating losses.

*Limitations and Future Perspectives* Ancestral reconstruction can be sensitive to the choice of taxa represented in the analysis. The species sample in this study is relatively small. Ctenophores were deliberately excluded to avoid convergence associated with very short branches. Xenacoelomorphans are also not represented in our data set. In addition, the number of genomes of unicellular Holozoa and Teretosporea that have been sequenced has increased substantially (Ruiz-Trillo et al. 2023). Incorporating these in a future study would give a better assessment of the pre-metazoan state.

Uncertainty in the species tree can contribute to downstream uncertainty. Early metazoan branching order is controversial (Najle et al. 2023; Whelan et al. 2017; Giribet and Edgecombe 2020; Laumer et al. 2018, 2019; Philippe et al. 2011; Simion et al. 2017; Dunn et al. 2008; Telford et al. 2015; Schultz et al. 2023; Simakov et al. 2020; Pandey and Braun 2020; Redmond and McLysaght 2021; Lartillot and Philippe 2008; Philippe et al. 2009). One issue is the placement of ctenophores relative to porifera. This uncertainty does not directly impact our results because ctenophores are not represented in this study. The placement of Trichoplax with respect to Cnidaria and Bilateria has also been called into question, with some analyses placing Trichoplax and Cnidaria as sister taxa (Simakov et al. 2020; Laumer et al. 2018). However, other recent work (Laumer et al. 2019; Simion et al. 2017; Najle et al. 2023) continues to support the topology used here (Figs. 1 and A1).

Missing protein domain annotations, genome assembly, and gene prediction errors are another source of noise. Proteins that contain domains that have not yet been characterized pose an even more fundamental problem. Fortunately, advances in AI-based protein structure prediction have dramatically increased both the size and the accuracy of protein domain resources (Paysan-Lafosse et al. 2025; Lau et al. 2024), offering exciting prospects for future studies.

Currently, there is no comprehensive functional annotation system for protein domains and this negatively impacted the depth of our functional analysis. Several projects currently underway will enable more comprehensive analyses of domain function in the not too distant future. The InterPro2GO project is a growing resource of manual annotation of domains with GO terms, based on experimental characterizations of the domain’s function (Burge et al. 2012; Blum et al. 2025). Complementing this effort, methodology to support automated mapping of Gene Ontology (GO) terms from proteins to domains is an active area of research (Weiner et al. 2008; Buchan and Jones 2020; Fang and Gough 2013; López and Pazos 2013; Ulusoy and Doğan 2024). More and better domain annotations will not only lead to more accurate reconstruction, but will also allow for more robust functional analyses.

This analysis focuses on the protein domain repertoire, defined to be the set of domains encoded in the genome without regard to how the domains are distributed across protein coding genes. Since protein domains are basic units of protein function, the set of protein domains encoded in a genome constitutes the functional toolkit available for building complex functions in that species. It is also important to study how the evolution of domain architectures has contributed to metazoan evolution. Domain architectures are larger, more complex and more prevalent in animals than in other branches of the Tree of Life (Tordai et al. 2005). Many families that are integral to complex multicellularity consist of multidomain proteins with complex and varied architectures (Patthy 2021). Very few studies have tackled phylogenetic comparative analyses of domain combinations in Metazoa (Zmasek and Godzik 2012; Grau-Bové et al. 2017). A growing body of work examines the history of domain rearrangements in a phylogenetic context (Buljan et al. 2010; Dohmen et al. 2020; Kersting et al. 2012; Cromar et al. 2014; Ekman et al. 2007; Gough 2005; Kummerfeld and Teichman. 2005; Forslund et al. 2019). This is an exciting, important, and challenging area for future work.

Probabilistic methods suffer from disadvantages including model violations and overfitting. Despite its flexibility, the Birth-Death-Gain model is challenged by very long running times, which depend on the computational cost of a single iteration and the number of iterations required to reach convergence. COUNT’s likelihood maximization procedure requires summing over many latent variables. It is not uncommon for latent variable models to have poorly defined, multimodal likelihood functions, which do not promote rapid convergence. It is difficult to predict how running times will scale as problem instances increase in size because the speed of convergence depends on the smoothness of the likelihood function in unexpected ways. Improvements in taxon sampling and domain architecture annotations are exciting directions for further study. At the same time, these will increase the scale of the problem. Happily, theoretical advances are keeping pace with new efficient algorithms for BDG likelihood calculations (Csűrös 2022).

Ongoing sequencing efforts are changing our understanding of the Metazoa-specific gene complement. Genes that are associated with metazoan phenotypes and were previously believed to be specific to Metazoa are increasingly found to have orthologs in close relatives to Metazoa, indicating that these families were already present in pre-metazoan ancestors. This suggests that the genesis of novel phenotypes may lie not in the acquisition of new genes, but in the co-option of existing genes to new functional roles. This raises questions about the utility of ancestral reconstruction of the protein repertoire, not just for this study but for all studies that use this approach. Changes that led to the phenotypic innovation seen in Metazoa may be due to regulatory or epigenetic changes, for example.

*Methodological challenges to principled ancestral reconstruction* Phylogenetic comparative analysis provides a powerful framework for investigating the genetic underpinnings of phenotypic change. Ancestral reconstruction of the genomic features can reveal the historical coincidence of genetic and phenotypic shifts, predict functional links, identify potential convergent evolution, and generate testable hypotheses. Phylostratigraphic analyses associate estimates of gene age with biological properties of interest. Present-day phyletic distributions have contributed to evidence for *de novo* gene origination. However, this compelling framework entails substantial methodological challenges.

First, relating changes in gene family composition to phenotypic evolution requires correct identification of corresponding genetic features in different genomes. Comparative analyses can be compromised by biased or sparse taxon sampling, gene prediction errors, bias in orthology databases, and failure to recognize orthologs due to remote homology (Natsidis et al. 2021; Moyers and Zhang 2015, 2017; Jain et al. 2019). This can be a problem for domains as well as genes (Kress et al. 2023; Nagy et al. 2011; Tassia et al. 2021).

Second, the assumptions underlying the ancestral reconstruction method must be aligned with the properties of the data set, including taxonomic and genomic characteristics and the idiosyncrasies of the underlying data. Dollo is just one of several parsimony methods that infer ancestral character states that minimize the number of state transitions, but differ in the relative importance of parallel gains, parallel losses, and reversals in resolving parsimony (Swofford and Maddison 1987). Probabilistic models of character evolution also embody specific assumptions about character evolution (Harmon 2019; Pagel 1999), although the dependence on model specifics may be underappreciated. Some accounts simply report that ancestral states were inferred using “maximum likelihood” and give the name of a software package, but do not provide a clear statement of the underlying model and its assumptions.

Phylogenetic reconciliation (Stolzer 2012), and related gene tree methods (e.g., Fernández and Gabaldón 2020), are a less widely used, but highly informative approach to inferring gene events and ancestral gene content. Parsimony-based reconciliation methods are computationally relatively efficient, yet are much less sensitive to the limitations of the parsimony assumption because the inference process is constrained by the information encoded in the gene tree topology. Phylogenetic reconciliation models can accommodate lateral gene transfer, allowing for multiple independent origins, albeit with an increase in computational complexity.

Whether parsimony or probability based, the quality of reconstruction will depend heavily on the extent to which the underlying assumptions of the model capture the properties of the biological system. Dollo parsimony is the algorithmic implementation of Dollo’s law of irreversibility, which posits that complex traits cannot be lost and regained in the same form. Whether these assumptions are always justified for complex traits is a long standing subject of debate (Elmer and Clobert 2025). In contrast, in genomic studies, the appropriateness of the Dollo model (or any other model) is less frequently discussed.

In the context of genomic features, Dollo’s law of irreversibility implies that a genomic feature cannot be gained more than once (Farris 1977). This assumption also underlies the phylostratigraphic approach to estimating gene age (Domazet-Lošo et al. 2007), which posits that the progenitor of a protein family arose in its cenancestor. When genetic features can be acquired horizontally, via lateral gene transfer (LGT) or introgression, for example, reconstruction paradigms that allow for multiple independent gains are required. This is particularly important in the face of increasing evidence for LGT in eukaryotes (Keeling 2024; Sibbald et al. 2020; Van Etten and Bhattacharya 2020). Dollo, which forbids multiple independent gains, tends to overestimate the both the age of a gene and the gene losses that occurred in the presence of horizontal gene flow (Capra et al. 2013).

In this study, analysis of the same data set with different ancestral reconstruction algorithms resulted in very different conclusions. With Dollo, domain loss is the dominant force: Losses exceed gains on two thirds of internal branches. With BDG, domain gain is the dominant force: Gains exceed losses on 75% of internal branches. This stark disagreement highlights the importance of selecting an ancestral reconstruction method with a compatible evolutionary model. Our results add to a growing body of work that suggests that the widespread use of phylostratigraphy and Dollo parsimony may be contributing to the perception that gene loss is a widespread phenomenon.

## Conclusions

Comparative phylogenetic analysis with a Birth-Death-Gain model reveals extensive and continual remodeling of the metazoan protein domain repertoire, suggesting an unexpected degree of plasticity. This is one of the few models capable of inferring instantaneous event rates and distinguishing between family-specific and branch-specific rates, providing a fresh perspective on the evolution of metazoan protein toolkit in relation to organismal innovation.

Evolution by genome streamlining, characterized by a dramatic increase in the ancestral genomic repertoire followed by genome reduction in multiple lineages, has been proposed as an important mode of genome evolution (Wolf and Koonin 2013; Albalat and Cañestro 2016). However, the evidence for genome streamlining obtained with a Birth-Death-Gain model in this analysis was quite modest. This may be due to the flexibility of Birth-Death-Gain models, which do not seek to minimize homoplasy, are agnostic with respect to gains versus losses, and allow for multiple independent origins of the same domain family. In contrast, analysis of the same data with the highly constrained Dollo parsimony model yielded a history dominated by losses. The stark contrast between these inferences prompts a critical reassessment of the methodologies commonly used in ancestral reconstruction.

## Supplementary Information

Below is the link to the electronic supplementary material.

Supplementary file 1 (pdf 2690 KB)

Supplementary file 2 (xlsx 8042 KB)
