# Supplementary material for: Evolution of the Metazoan Protein Domain Repertoire Revealed by a Birth-Death-Gain Model
Source: J Mol Evol. 2025 Dec 29;93(6):777–99. doi: 10.1007/s00239-025-10286-0 (PMC12756402; doi:10.1007/s00239-025-10286-0)
Supplement: Supplementary file 1 — (pdf 2690 KB) [file 239_2025_10286_MOESM1_ESM.pdf]

Appendix A Supplementary Materials

Supplementary Figures

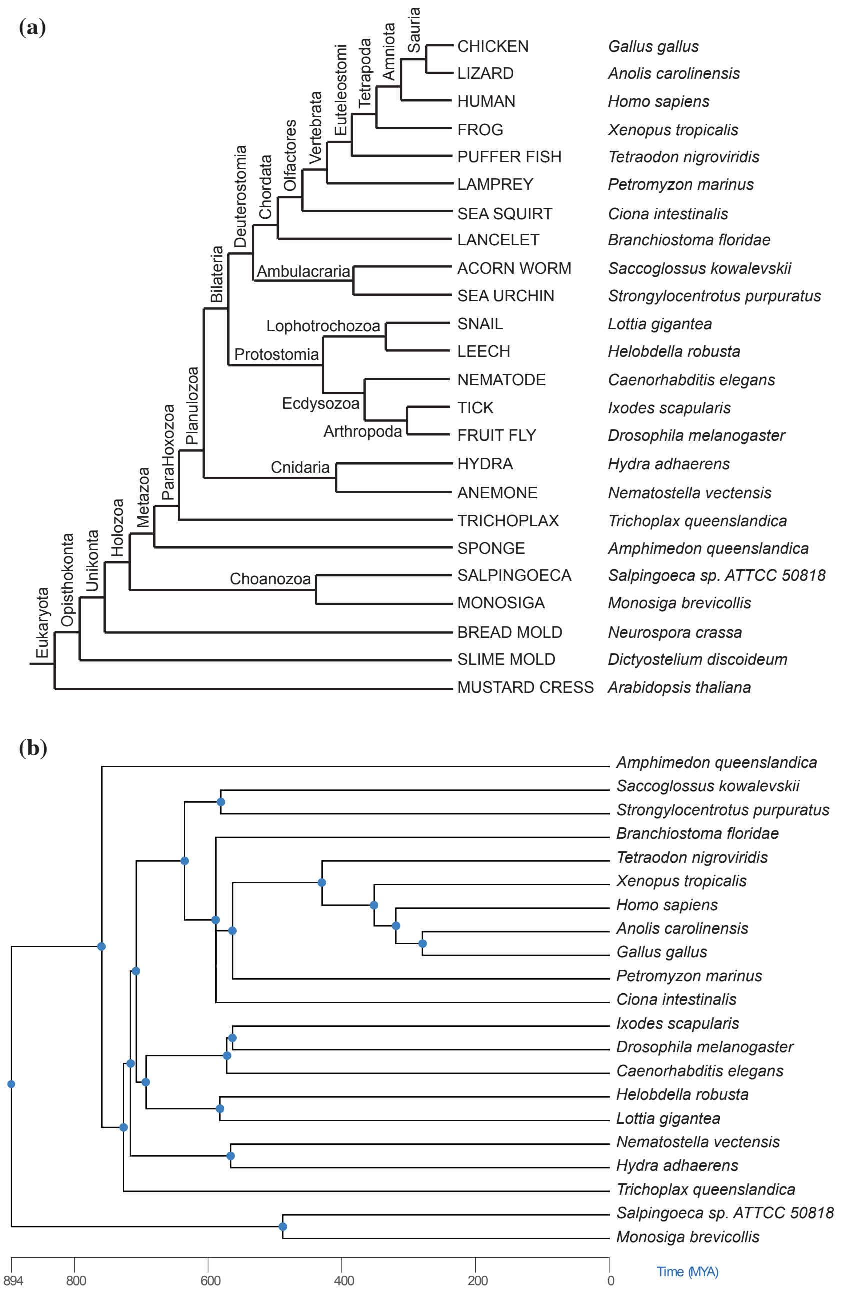

Fig. A1: **(a)** Cladogram of phylogenetic relationships between 21 holozoa and 3 outgroup species, adapted from Philippe et al. (2009). Common names of present-day species provided in all caps; scientific binomial nomenclature on the right. **(b)** Dated species tree for the 21 holozoan species studied in this work, obtained with TimeTree5 (Kumar et al., 2022).

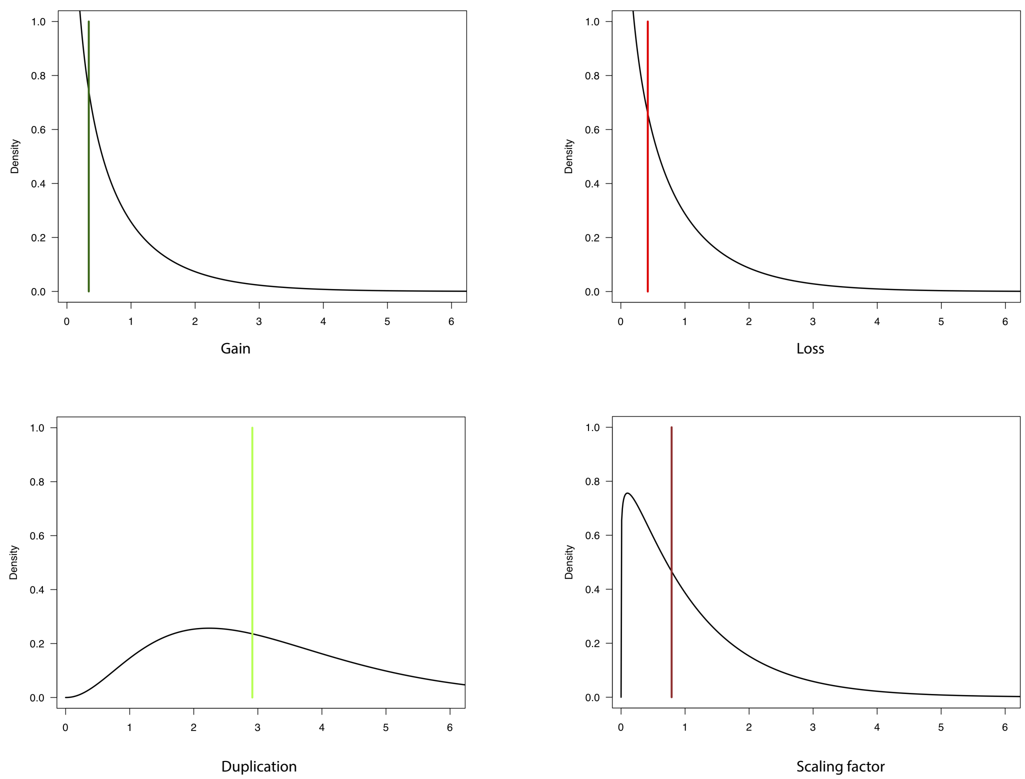

Fig. A2: Discretized gamma distribution with two bins for family-specific event rates. Vertical lines indicate the separation between the slow and fast bins.

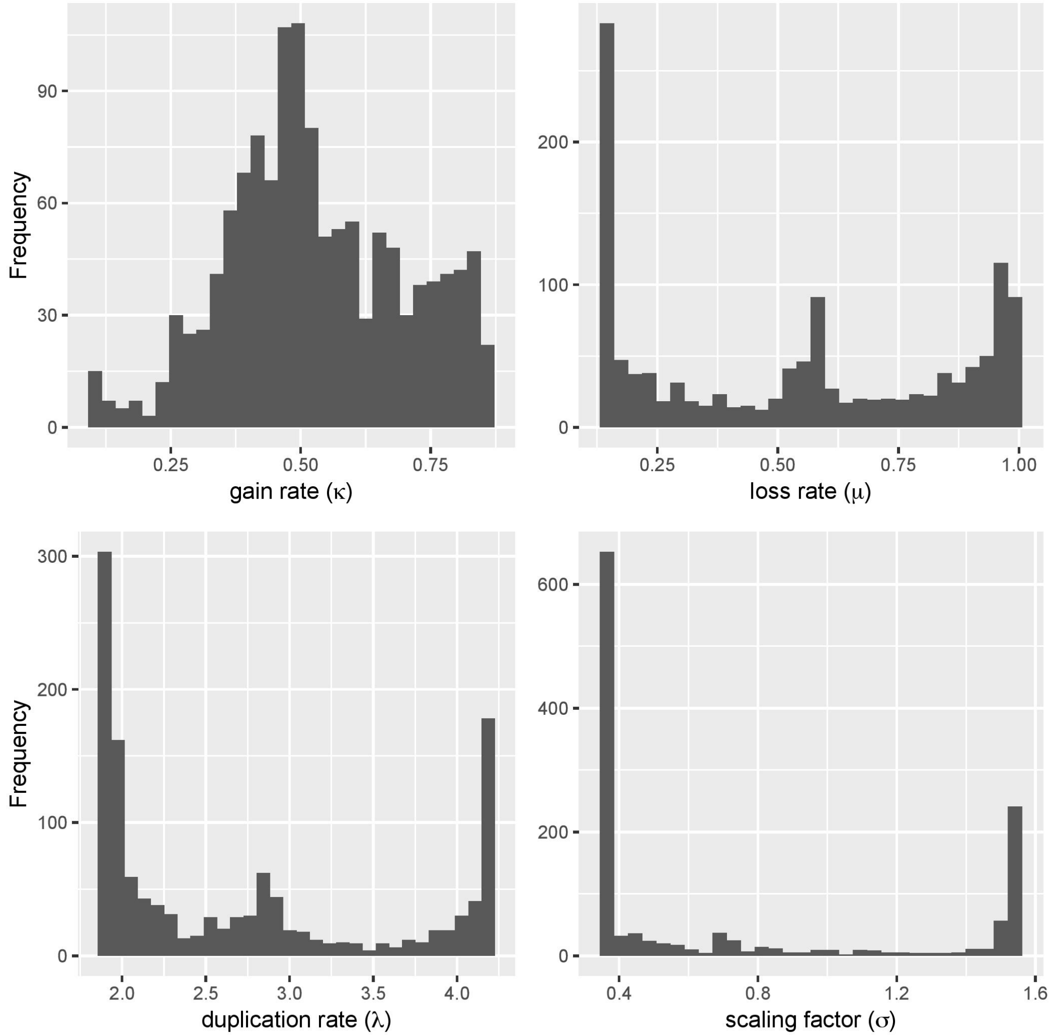

Fig. A3: Histograms of expected family-specific rates for the 1283 Holozoan domain families in our data set.

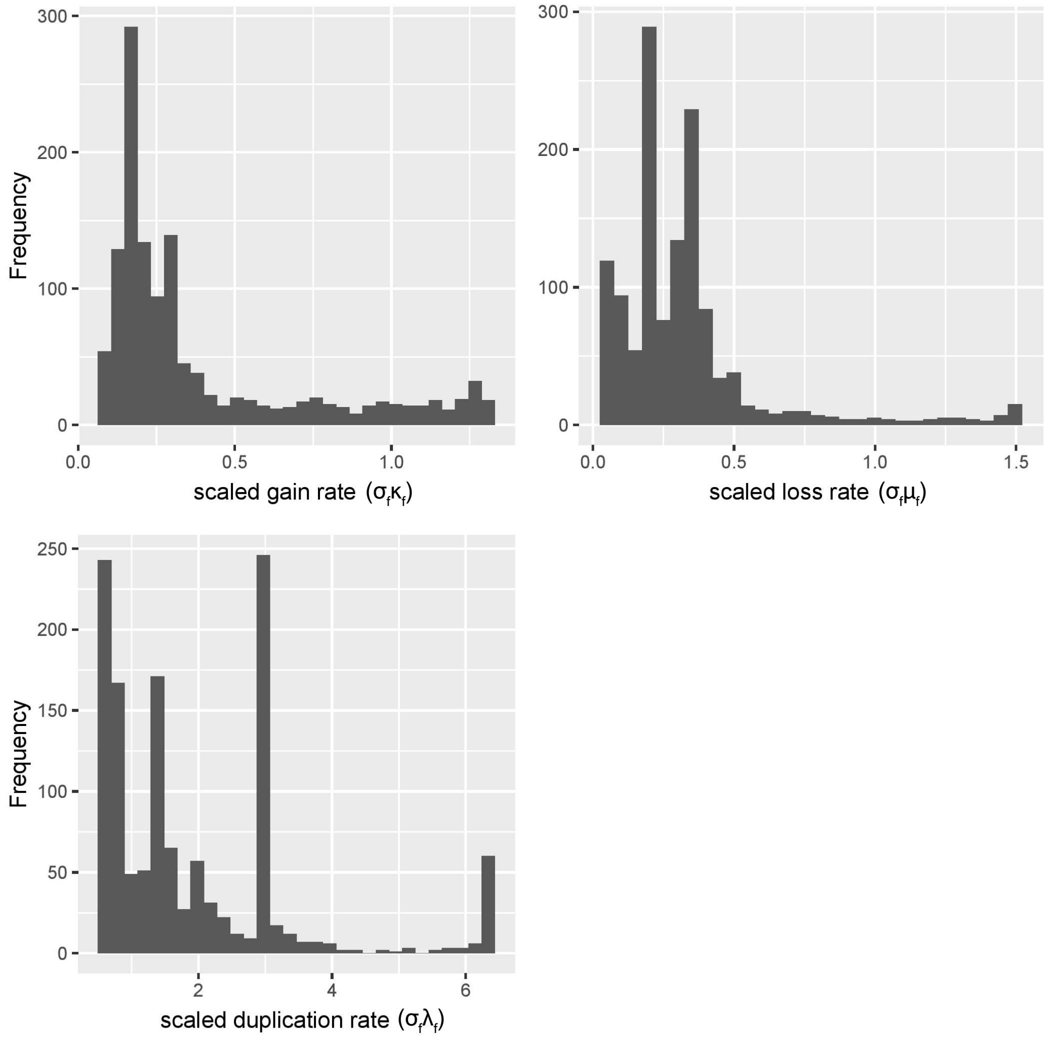

Fig. A4: Distributions of expected domain family rates, scaled by scaling factor. The scaled expected rate of each domain family was calculated by multiplying the expected family rate by the expected family scaling factor.

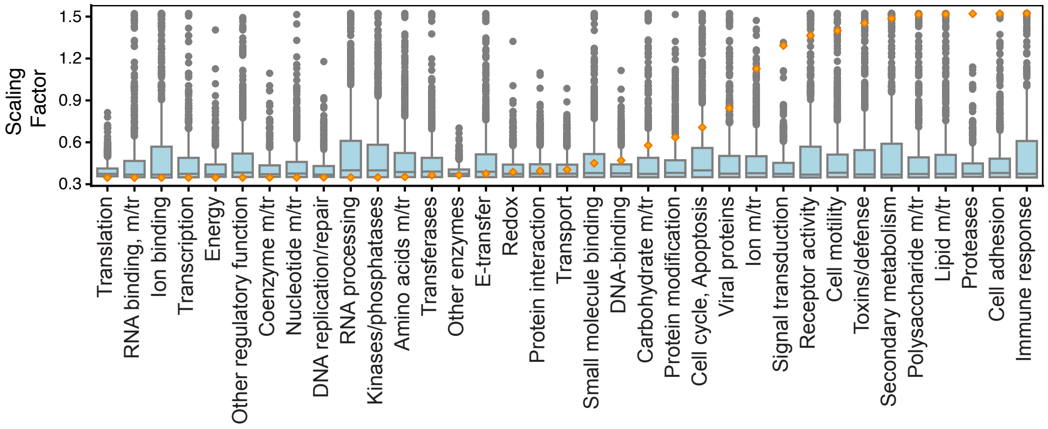

Fig. A5: Box-and-whisker plots showing the distributions of medians across functional categories for scaling factor (σ_f) and scaled duplication ($\sigma_f \lambda_f$), loss ($\sigma_f \mu_f$), and gain ($\sigma_f \kappa_f$) over 1,000 simulations. Categories are ordered by ascending median scaling factor value in the genuine data. For each rate category, orange points indicate the median in the genuine data. Abbreviation: m/tr = metabolism and transport.

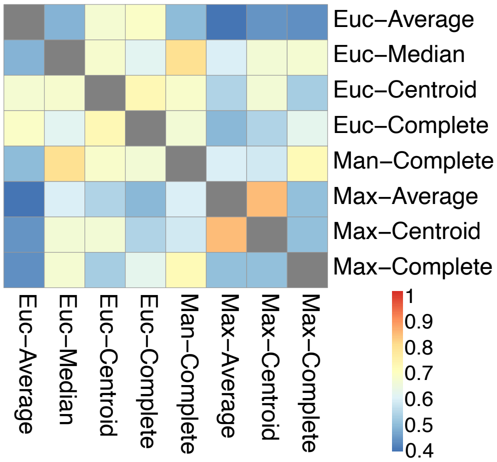

Fig. A6: Normalized Mutual Information (NMI) comparison of significant ($p < 0.05$) clusterings obtained using Statistical Hierarchical Clustering. Only clusterings with 3 or more clusters are shown. NMI provides an assessment of clustering similarity. Pairs of clusterings with NMI values greater than 0.6 are considered to have good aggrement between clusters, where NMI values greater than 0.8 are considered to have substantial overlap in the information they encode. All diagonal cells are masked in grey. Metric abbreviated as follow: Euc (Euclidean), Max (Maximum); Man (Manhattan distance).

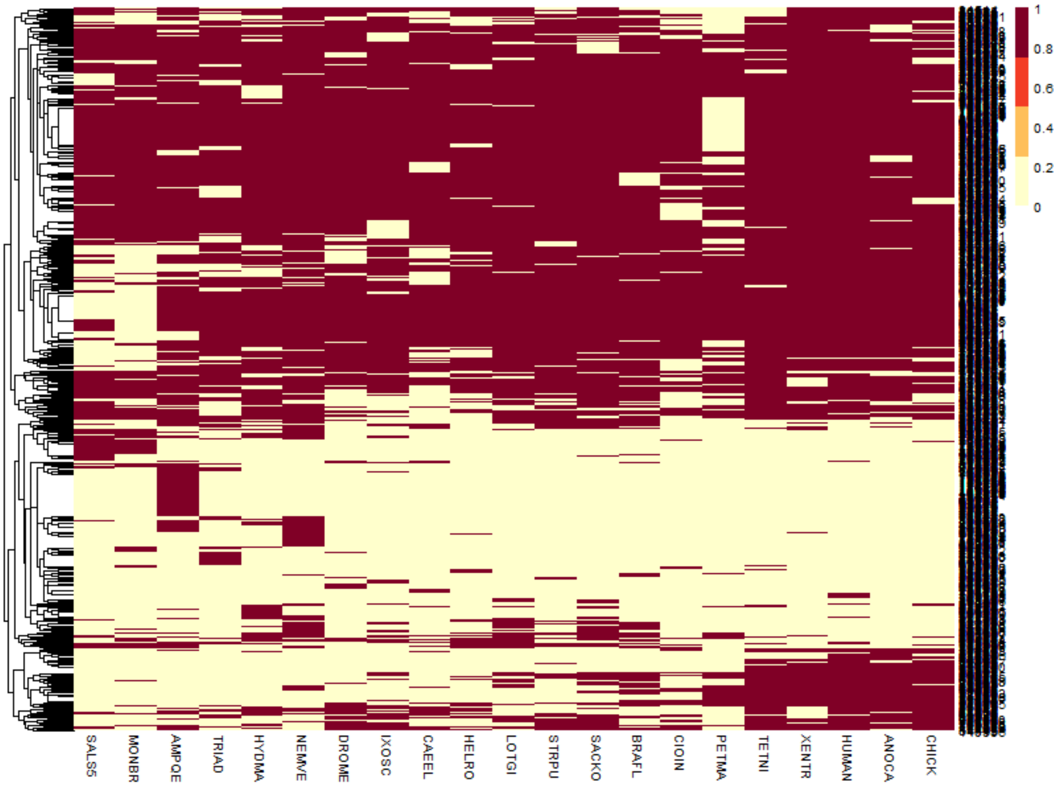

Fig. A7: Distribution of the 782 non-core domain families encoded in 21 holozoan genomes (dark red: present; yellow: absent). Rows represent domain families, and columns represent present-day species. Dendrogram of domain family phylogenetic profiles generated using the pheatmap package in R (Kolde, 2018).

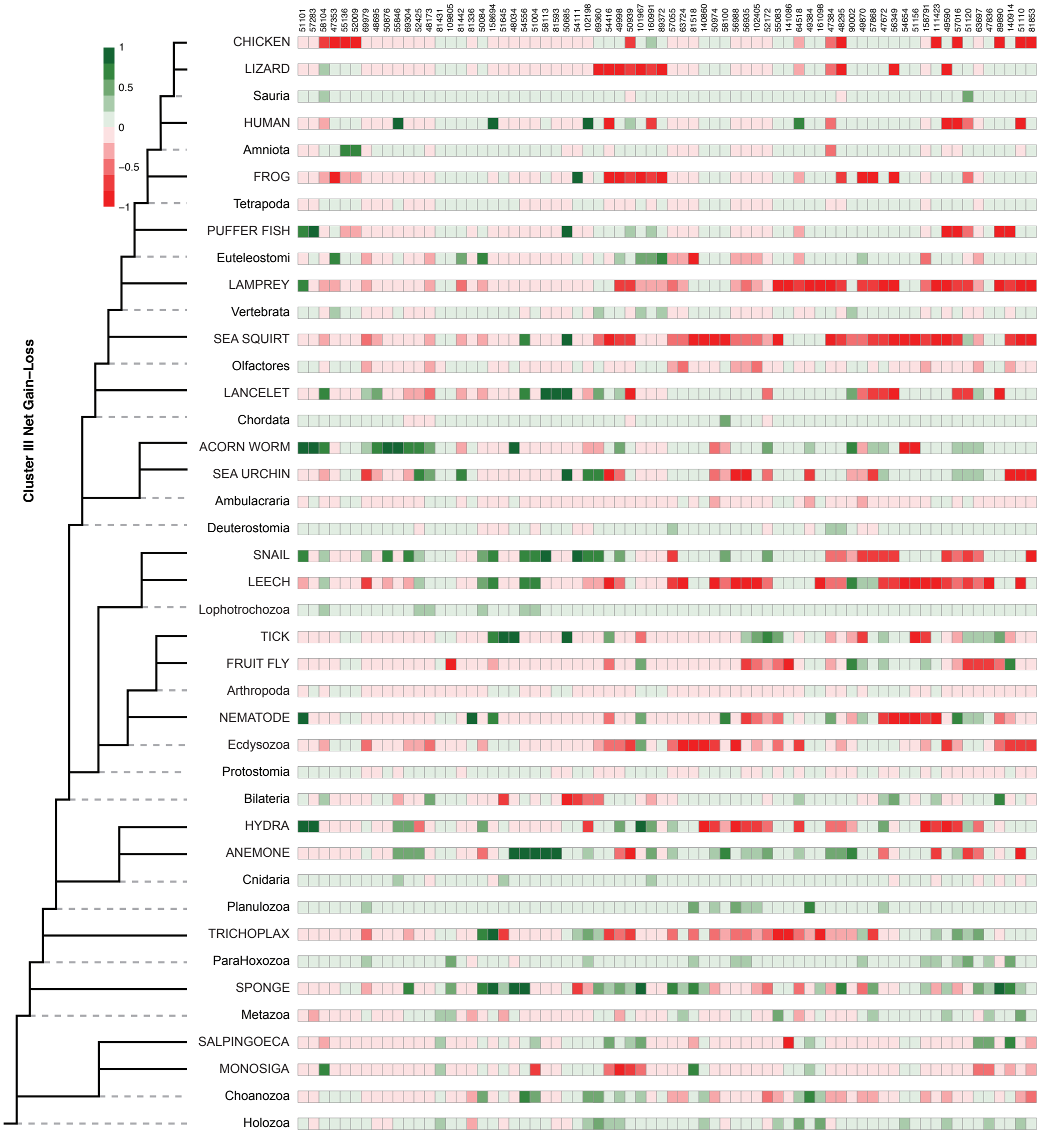

Fig. A8: Probability of family gain minus probability of family loss on each branch of the species tree for the 70 domain families in rate Cluster III. Clusters are numbered as in Figure 6. A family f is considered to have a high-confidence gain event (respectively, a high-confidence loss event) on branch b if the net probability is greater than 0.6 (respectively, less than -0.6).

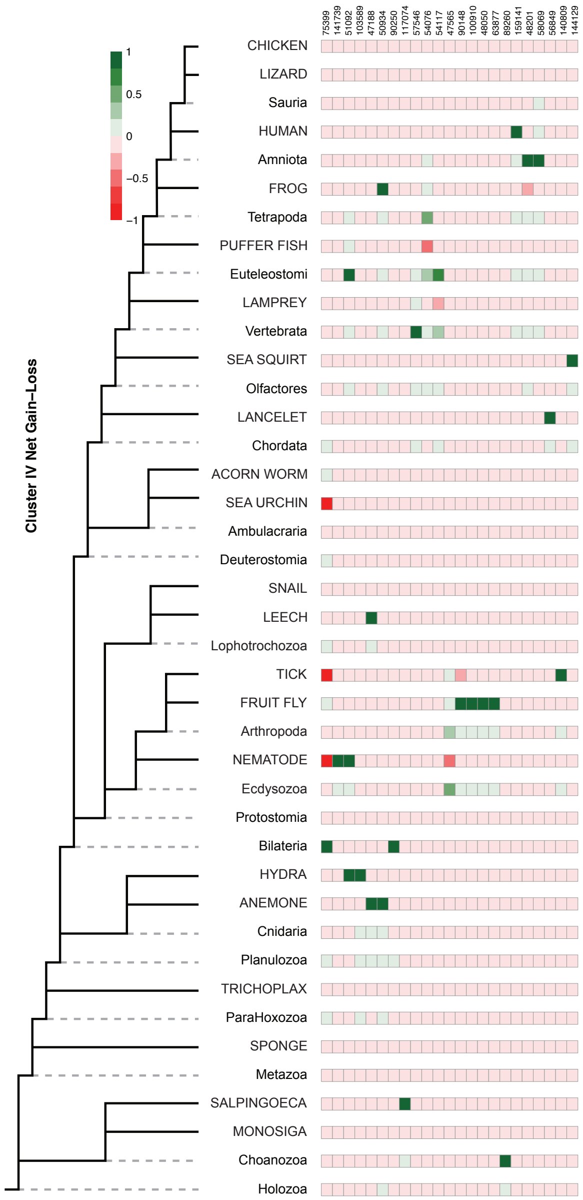

Fig. A9: Probability of family gain minus probability of family loss on each branch of the species tree for the 23 domain families in rate Cluster IV. Clusters are numbered as in Figure 6. A family f is considered to have a high-confidence gain event (respectively, a high-confidence loss event) on branch b if the net probability is greater than 0.6 (respectively, less than -0.6).

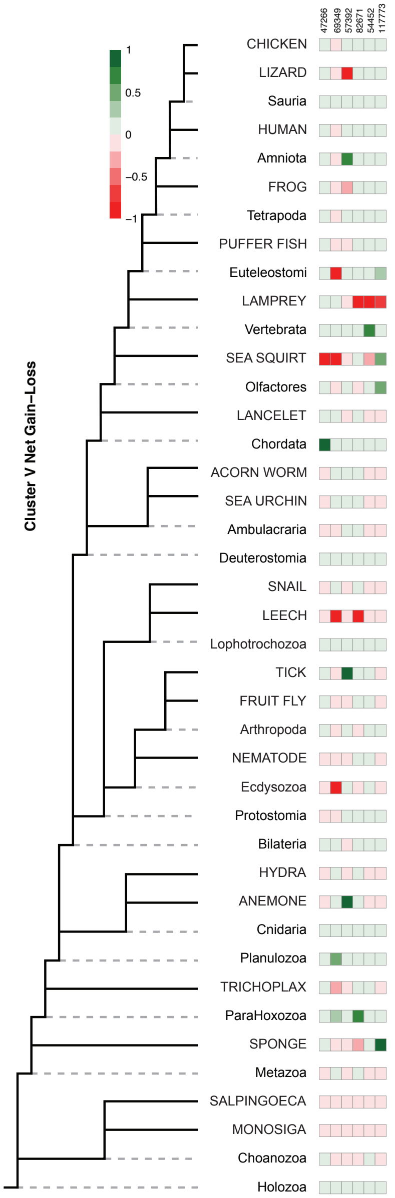

Fig. A10: Probability of family gain minus probability of family loss on each branch of the species tree for the 6 domain families in rate Cluster V. Clusters are numbered as in Figure 6. A family f is considered to have a high-confidence gain event (respectively, a high-confidence loss event) on branch b if the net probability is greater than 0.6 (respectively, less than -0.6).

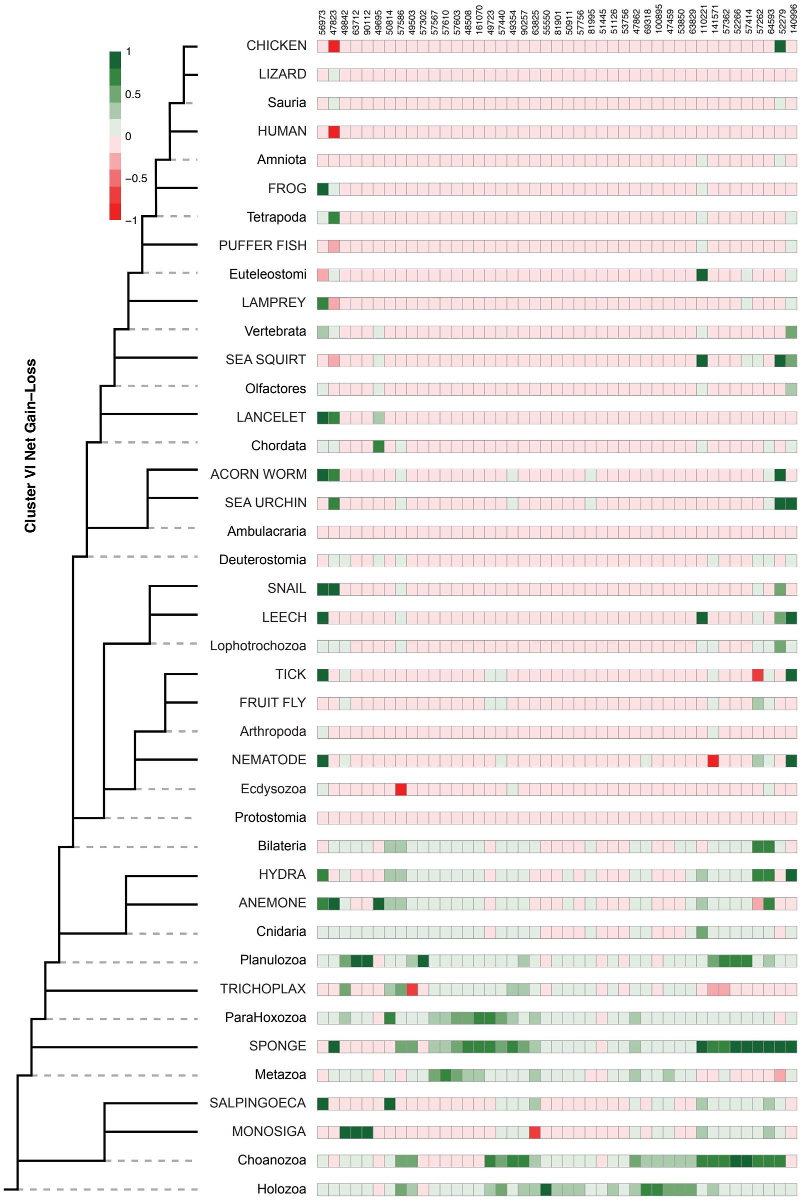

Fig. A11: Probability of family gain minus probability of family loss on each branch of the species tree for the 43 domain families in rate Cluster VI. Clusters are numbered as in Figure 6. A family f is considered to have a high-confidence gain event (respectively, a high-confidence loss event) on branch b if the net probability is greater than 0.6 (respectively, less than -0.6).

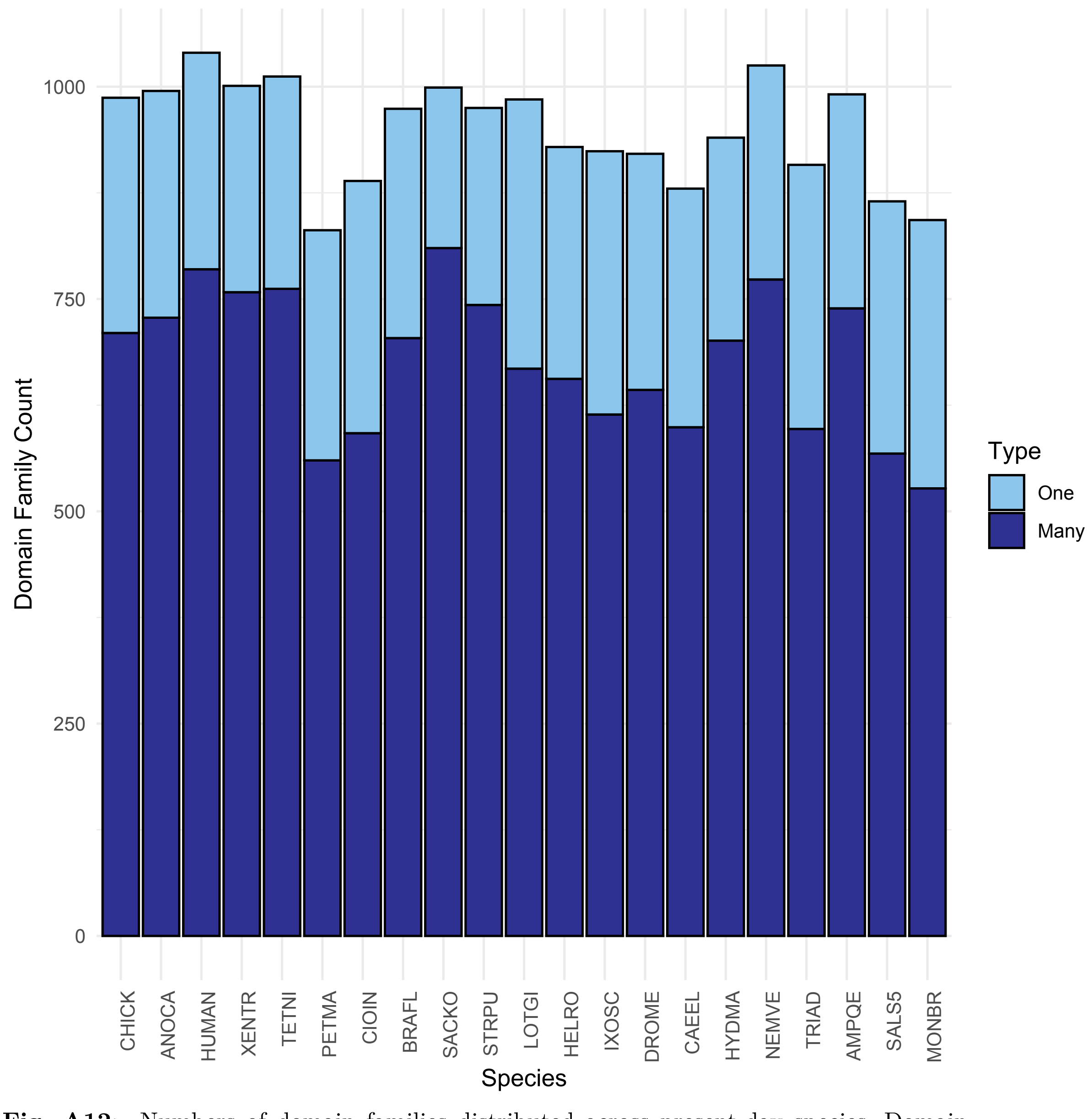

Fig. A12: Numbers of domain families distributed across present-day species. Domain families present in a single copy and present in multicopy are shown in light blue and dark blue, respectively. Species names abbreviated as in Figures 7.

Supplementary Tables

Metric	Linkage	Clusters	Mean Silhouette	Actual p-value
Euclidean	Average	12	0.51	1.02E-03
	Median	6	0.59	2.35E-03
	Centroid	6	0.58	2.82E-17
	Complete	10	0.57	1.40E-02
Manhattan	Average	2	0.60	7.43E-13
	Centroid	2	0.59	1.57E-18
	Complete	4	0.60	2.16E-15
Maximum	Average	5	0.56	1.51E-07
	Centroid	5	0.58	2.95E-18
	Complete	3	0.57	6.73E-07

Table A1: Overview of the 10 significant clusterings of scaled event rates ($p < 0.05$).

SUPERFAM ID	Domain Family	Copies in 24 species Total	Mean	Max
48403	Ankyrin repeat	7307	304.46	1545
48452	TPR-like	6780	282.50	916
48726	Immunoglobulin	18695	778.96	3527
49265	Fibronectin type III	9386	391.08	2237
49313	Cadherin-like	8851	368.79	997
49562	C2 domain (Calcium/lipid-binding domain, CaLB)	3519	146.63	334
49785	Galactose-binding domain-like	3327	138.63	642
49854	Spermadhesin, CUB domain	3486	145.25	623
49899	Concanavalin A-like lectins/glucanases	5312	221.33	625
50044	SH3-domain	3659	152.46	363
50156	PDZ domain-like	3776	157.33	367
50729	PH domain-like	6376	265.67	641
50978	WD40 repeat-like	6755	281.46	553
51735	NAD(P)-binding Rossmann-fold domains	5081	211.71	482
52058	L domain-like	6594	274.75	1083
52540	P-loop containing nucleoside triphosphate hydrolases	26188	1091.17	2436
52833	Thioredoxin-like	3718	154.92	365
53098	Ribonuclease H-like	3343	139.29	683
53335	S-adenosyl-L-methionine-dependent methyltransferases	3781	157.54	429
53474	alpha/beta-Hydrolases	3863	160.96	450
54236	Ubiquitin-like	3532	147.17	377
54928	RNA-binding domain, RBD	6223	259.29	766
56112	Protein kinase-like (PK-like)	12341	514.21	1409
56436	C-type lectin-like	4289	178.71	920
56487	SRCR-like	4031	167.96	1336
57184	Growth factor receptor domain	5609	233.71	846
57196	EGF/Laminin	15364	640.17	3261
57424	LDL receptor-like module	5295	220.63	1261
57535	Complement control module/SCR domain	5202	216.75	932
57667	beta-beta-alpha zinc fingers	31637	1318.21	6393
57716	Glucocorticoid receptor-like (DNA-binding domain)	4206	175.25	360
57850	RING/U-box	7657	319.04	863
81296	E set domains	4507	187.79	393
81321	Family A G protein-coupled receptor-like	9702	404.25	1193
82895	TSP-1 type 1 repeat	4072	169.67	453
103473	MFS general substrate transporter	4982	207.58	423

Table A2: The 41 families with more than 3200 instances across all species and unable to process by COUNT in Pass 1. 28 domain families were multicopy in all species. 39 domain families (48726 and 56487 exceptions) were multicopy in all species except the outgroups.

SUPERFAM ID	Domain Family	Copies in 24 species Total	Mean	Max
47113	Histone-fold	2237	93.21	385
47986	DEATH domain	2347	97.79	648
48264	Cytochrome P450	2396	99.83	288
49599	TRAF domain-like	1554	64.75	442
50494	Trypsin-like serine proteases	1888	78.67	272
52047	RNI-like	2861	119.21	398
52200	Toll/Interleukin receptor TIR domain	1129	47.04	293
53300	vWA-like	2527	105.29	244
53822	Periplasmic binding protein-like I	1499	62.46	385
54695	POZ domain	3093	128.88	500
55486	Metalloproteases (zincins), catalytic domain	2607	108.62	337
56219	DNase I-like	2131	88.79	620
56496	Fibrinogen C-terminal domain-like	1373	57.21	271
56672	DNA/RNA polymerases	2521	105.04	709
57256	Elafin-like	884	36.83	663
57625	Invertebrate chitin-binding proteins	796	33.17	284
57845	B-box zinc-binding domain	1865	77.71	351
81324	Voltage-gated potassium channels	2931	122.12	283
101898	NHL repeat	1167	48.62	382
109640	KRAB domain (Kruppel-associated box)	585	24.38	423
117281	Kelch motif	2077	86.54	418
141072	CalX-like	1226	51.08	260

Table A3: Large domain families that COUNT failed to process in Pass 2, emitting NaN values as results rather than parameter estimates. These families were excluded from downstream analyses.

Node name	Branch-specific parameters		
	t_b	λ_b	κ_b
CHICKEN	0.362	1.320	0.032
LIZARD	0.250	2.280	0.005
Sauria	2.74E-04	54.000	33.500
HUMAN	0.083	8.600	0.436
Amniota	0.008	12.000	2.090
FROG	0.181	4.010	0.052
Tetrapoda	0.007	7.720	2.030
PUFFER FISH	0.193	4.550	0.167
Euteleostomi	0.087	3.910	1.220
LAMPREY	1.270	0.630	0.014
Vertebrata	0.030	10.200	2.140
SEA SQUIRT	0.739	1.370	0.059
Olfactores	0.035	5.140	0.419
LANCELET	0.172	9.390	0.396
Chordata	4.24E-04	2.25E-05	97.600
ACORN WORM	0.226	7.490	0.236
SEA URCHIN	0.219	9.720	0.636
Ambulacraria	0.022	4.060	1.02E-05
Deuterostomia	0.001	5.390	39.200
SNAIL	0.239	5.700	0.355
LEECH	0.628	2.250	0.091
Lophotrochozoa	1.34E-04	4.39E-05	97.500
TICK	0.449	2.700	0.200
FRUIT FLY	0.272	4.140	0.140
Arthropoda	0.025	3.810	0.101
NEMATODE	0.630	2.210	0.072
Ecdysozoa	0.257	0.017	0.091
Protostomia	0.003	9.280	1.02E-05
Bilateria	0.089	0.043	1.150
HYDRA	0.608	2.080	0.250
ANEMONE	0.204	6.430	1.380
Cnidaria	0.006	35.500	5.880
Planulozoa	0.001	2.140	97.700
TRICHOPLAX	0.501	2.350	0.221
ParaHoxozoa	0.009	13.900	12.900
SPONGE	0.362	5.170	1.080
Metazoa	0.113	0.132	1.020
SALPINGOECA	0.250	3.040	0.241
MONOSIGA	0.441	1.490	0.125
Choanoflagellida	0.296	2.000	0.398
Holozoa	0.035	8.270	3.750

Event	Family-specific parameters		
	**Shape**	**Slow**	**Fast**
Birth (λ_f)	3.240	1.920	4.210
Gain (κ_f)	0.628	0.098	0.855
Death (μ_f)	0.714	0.136	0.982
Scale (σ_f)	1.100	0.347	1.520

Table A4: Parameter values inferred by COUNT's maximum likelihood estimation procedure. Branch-specific parameters: gain rate (κ_b), birth rate (λ_b), and duration (t_b) of the evolutionary process on each branch. Branch-specific loss rates are set to 1 by normalization. Family-specific parameters: Gamma shape parameter and midpoint values for the slow and fast rate categories for each of the four family-specific parameters $\{\sigma_f, \kappa_f, \lambda_f, \mu_f\}$. The mean of the Poisson distribution describing family sizes in the root of the species tree, $\phi = 0.80$.

Species	Number of families
SPONGE	37
NEMATODE	4
SEA SQUIRT	1
FRUIT FLY	5
LEECH	2
HUMAN	6
HYDRA	3
TICK	3
SNAIL	1
MONOSIGA	2
ANEMONE	9
ACORN WORM	1
SALPINGOECA	2
TRICHOPLAX	8

Table A5: Number of species-specific domain families in present-day species.

Clade	Total	Clusters					
		**I**	**II**	**III**	**IV**	**V**	**VI**
All (24)	445	310	122	1	0	0	12
Holozoa (21)	501	336	146	1	0	0	18
Metazoa (19)	547	359	163	1	0	0	24
Bilateria (15)	594	382	176	1	1	0	34
Euteleostomi (5)	899	640	194	16	5	4	40

Table A6: Core family representation across rate clusters: The core set for a given clade consists of all families that are present in every present-day repertoire in the clade, within our data set. Numbers in parentheses are the number of present-day species included in each set. The second column shows the total number of domain families in each core set. Columns **Cluster I - Cluster VI** shows the number of core domain families in the present-day species of each core set and each cluster.

Species	Met	Inf	IC	Reg	Gen	O	EC
CHICKEN	318	146	145	128	80	84	37
LIZARD	325	146	145	132	78	86	33
HUMAN	339	154	146	135	84	91	37
FROG	324	152	145	132	80	82	35
PUFFER FISH	339	148	142	133	79	81	37
LAMPREY	262	126	131	113	65	63	30
SEA SQUIRT	293	136	134	118	68	64	28
LANCELET	331	138	140	128	75	76	32
ACORN WORM	338	149	147	129	70	83	29
SEA URCHIN	329	148	143	126	71	75	31
SNAIL	328	149	148	126	69	82	31
LEECH	313	143	143	119	67	71	27
TICK	298	135	137	118	58	69	25
FRUIT FLY	300	142	136	128	62	79	29
NEMATODE	300	147	140	125	66	72	26
HYDRA	356	150	145	131	72	83	30
ANEMONE	316	147	141	123	66	72	28
TRICHOPLAX	295	150	143	119	64	67	26
SPONGE	344	154	140	126	61	77	27
SALPINGOECA	306	135	128	93	58	63	20
MONOSIGA	307	142	131	103	53	67	19
HUMAN : MONOSIGA	1.11	1.14	1.14	1.45	1.45	1.44	1.85

Table A7: The general functional category distribution across the 1999 holozoan domain families in our data set that have functional annotations. The last row gives the ratio, for each functional category, of the number of domains in that category encoded in the human and *Monosiga brevicollis* genomes. Met: Metabolism; Inf: Information; IC: Intra-cellular processes; Reg: Regulation; Gen: General; O: Other; EC: Extra-cellular processes.

General	Detailed	I	II	III	IV	V	VI	Total
Extra-cellular	Cell adhesion	9	9	1	0	1	2	22
	Immune response	1	3	0	1	2	2	9
	Blood clotting	0	1	0	0	0	1	2
	Toxins/defense	6	0	3	2	1	1	13
General	Small molecule binding	13	2	3	0	0	0	18
	Ion binding	7	1	1	0	0	0	9
	Lipid/membrane binding	1	0	0	0	0	0	1
	Ligand binding	3	0	0	0	0	0	3
	General	14	5	1	0	0	0	20
	Protein interaction	28	9	1	1	0	1	40
	Structural protein	3	0	0	1	0	1	5
Infor-mation	Chromatin structure	4	1	0	0	0	0	5
	Translation	86	2	0	0	0	0	88
	Transcription	21	0	0	0	0	0	21
	DNA replication/repair	38	6	3	0	0	0	47
	RNA processing	8	1	0	1	0	0	10
Intra-cellular	Cell cycle, Apoptosis	8	6	0	0	0	0	14
	Phospholipid m/tr	2	4	0	0	0	0	6
	Cell motility	8	6	2	1	0	1	18
	Protein modification	18	7	0	0	0	3	28
	Proteases	15	15	4	0	0	5	39
	Ion m/tr	12	7	0	0	0	3	22
	Transport	34	9	2	0	0	1	46
	Trafficking/secretion	2	0	0	0	0	0	2
Metabolism	Energy	44	1	5	1	0	0	51
	E- transfer	15	1	1	1	0	0	18
	Photosynthesis	3	0	0	0	0	0	3
	Amino acids m/tr	16	2	0	0	0	0	18
	Nitrogen m/tr	0	1	0	0	0	0	1
	Nucleotide m/tr	21	6	1	0	0	0	28
	Carbohydrate m/tr	14	2	2	0	0	3	21
	Polysaccharide m/tr	6	2	4	1	0	2	15
	Coenzyme m/tr	43	3	3	0	0	0	49
	Lipid m/tr	6	4	1	0	0	2	13
	Cell envelope m/tr	2	0	0	1	0	0	3
	Secondary metabolism	4	4	1	0	0	0	9
	Redox	37	6	4	0	0	0	47
	Transferases	17	6	1	0	0	0	24
	Other enzymes	96	19	8	0	0	2	125
Other	Unknown function	88	5	5	3	0	6	107
	Viral proteins	11	0	2	2	1	1	17
Regulation	RNA binding, m/tr	19	2	0	0	0	0	21
	Receptor activity	5	4	1	1	0	0	11
	DNA-binding	37	13	1	0	1	2	54
	Kinases/phosphatases	10	1	1	0	0	0	12
	Signal transduction	20	14	3	2	0	3	42
	Other regulatory function	19	2	1	0	0	0	22
Total		874	192	66	19	6	42	1199

Table A8: Distribution of the detailed functions defined by Vogel et al. (2005) across the rate clusters inferred in this work. The functions highlighted in gray were deemed uninformative and excluded from functional analyses. “Viral proteins” is the only informative functional annotation in the “Other” general category. In addition, 84 domain families with no annotation are distributed across clusters as follows: Cluster I: 71; Cluster II: 1; Cluster III: 4; Cluster IV: 4; Cluster V: 4; Cluster VI: 0.

**General Function**	**I**	**II**	**III**	**IV - VI**
Extra-cellular processes	**1.89e-06**	0.563	4.050	**5.69e-06**
General	2.950	3.460	4.940	5.810
Information	**1.23e-10**	**6.42e-05**	0.168	0.010
Intra-cellular processes	**1.82e-05**	**7.44e-06**	3.460	1.030
Metabolism	0.068	0.075	0.454	0.060
Regulation	0.938	0.404	3.320	4.110
Viral	4.520	1.150	5.060	0.262

Table A9: Significance of the distribution of general functions (Vogel et al., 2005) across scaled rate clusters. "Viral" indicates families assigned to only informative functional annotation in the "Other" general category. Bold: $p < 0.05$ (hypergeometric test with multiple testing correction).

Supplementary Data

- Genome identifiers (sheet 1)
- Input profiles (sheet 2)
- Expected states and events on tree(sheet 3)
- Expected states and events for families (sheet 4)
